# Adaptive overcurrent protection considering fault current limiters effect

**DOI:** 10.1038/s41598-025-05135-5

**Published:** 2025-06-20

**Authors:** R. A. Mahmoud, K. G. Ghaly, S. A. Hasan

**Affiliations:** 1 Electrical Power and Machines Engineering (PME) Department, College of Engineering Science & Technology, Misr University for Science and Technology (MUST), 6th of October City, Giza Egypt; 2 Electrical Power and Machines Department, Faculty of Engineering, Helwan University, Helwan, Cairo Egypt

**Keywords:** Power networks, distributed generators (DGs), Fault current limiters (FCLs), Conventional overcurrent relays (COCRs), Adaptive overcurrent relays (AOCRs), Operating time (OPT), Energy science and technology, Engineering

## Abstract

Due to the rise in power consumption in recent years, the rated capacity of the power system has increased, resulting in an increase in the presence of Distributed Generators (DGs) in electrical networks. As a result, short-circuit currents surge when shunt faults occur. Fault Current Limiters (FCLs) are an effective way to suppress fault currents in the power systems. On the other hand, FCLs have an impact on the response speed of the protective devices, such as over-current relays (OCRs), which increase the relay operating time, raising the electrical and mechanical stresses on the system equipment. This paper presents an adaptive OCR algorithm considering the FCLs effect without any delay time. The proposed algorithm includes two modules: (1) a Z-score algorithm based on both the mean and the standard deviation values of the input current data, which is used to detect fault conditions, and (2) tripping characteristic curves based on the current Mean Ratio, which are applied to estimate the appropriate operating time of the adaptive OCR. To verify the method performance, a power system with real parameters is simulated on the Alternative Transient Program platform, and the algorithm procedure is implemented in the MATLAB program. Extensive simulation studies of load changes and various fault types are conducted, encompassing a wide range of fault initiation angles, fault resistances, and fault zones. The quantitative findings of these studies are analyzed in the presence and absence of FCLs/DGs. The simulation results indicate that the proposed algorithm can operate online and adjust its operating time settings automatically. As a consequence, it is able to detect fault instances upon which the relay sends a tripping flag, yet remains inactive under normal operating conditions. The algorithm speed and sensitivity are controllable using a moving data window size. Moreover, it is characterized by being easy to use, reliable, and accurate. Furthermore, the Z-score of the phase current can be used to identify the faulty phase and classify the fault type. In addition, the algorithm can be integrated with other digital protection and automation systems to be applied in conventional and smart grids.

**Supplementary Information:**

The online version contains supplementary material available at 10.1038/s41598-025-05135-5.

##  Introduction

As a result of increasing electricity demand, the distribution systems are continuously expanding. At certain locations in the electrical distribution system, this growth may result in greater fault current levels^1^. However, as power-producing facilities have grown and renewable energy resources have been introduced, power transformer ratings have been exceeded. One of these countermeasures is the replacement of smaller power transformers with larger ones. Due to the lower impedance of the largest power transformers, the fault current can be increased. The increase in fault current may surpass the power equipment’s cut-off ratings^2^. This may necessitate a switchgear update with stronger short-circuit ratings. However, updating the switchgear may not be feasible due to the following: (I) a high initial cost, and (II) a reduction in system reliability during the construction period. Short-circuit currents have a lot of energy and can cause damage to the electrical equipment. When a fault occurs, circuit breakers often have a reaction time delay that enables the fault current to pass for nearly two or three cycles of the fundamental frequency before its operation. However, circuit breakers occasionally fail to deal with the high strength of fault currents, preventing the system from crashing^3^. Various approaches were applied as solutions to reduce the high fault current, including the installation of series reactors or the replacement of either high impedance transformers or circuit breakers with larger power capacity. However, these approaches have several drawbacks, such as the possibility of power loss and voltage drop during normal operation^4^. One of the most successful strategies for avoiding these issues is to restrict the fault current to a reasonable level, as recommended by the Fault Current Limiter (FCL).

### The importance of the FCL in power systems

The FCL is an electrical element, connected in series with power lines to limit the fault current to an acceptable level, while it provides low impedance and power loss under normal operating conditions^5,6^. When the fault occurs on any component of the electric power grid, the functional role of the FCL is to control short-circuit current by swiftly adding equivalent current limiter impedance. Recently, the fault current limiter has been observed as an effective current limiting solution for the short-circuit current in these conditions, and many types of FCLs have been created^7,8^. Due to the connection of DGs, whose access capability might lead to a greater fault current level, current limiting elements were becoming increasingly relevant. High electrical resistivity characterizes the quenched state, which is employed to keep the initial peak of the fault current within rated operational limits^9^. In^10,11^, the major aim of these studies concentrates on the optimum location of the FCL in the power systems. The FCL can combine the sensing, triggering, and current limiting functions into one element. It has rapid reaction time and automatic recovery capabilities that other current-limiting elements lack^12,13^. This can successfully switch off the circuit breaker of the feeder from its malfunction, as well as protect the electrical equipment. It merely suppresses the fault current during the first half-cycle from the fault inception time. It performs better than the circuit breakers and relays because the circuit breakers require at least 2–3 cycles to operate^14^. In addition, it restricts the large fault currents. The FCL provides various other benefits to the power systems, including (I) improving power system stability, (II) reducing voltage sag during the fault time and after the fault clearance, and (III) saving costs for new electrical installations^15^. The utilization of the FCL may have a favorable influence on power system operation. The FCL can increase voltage quality by decreasing voltage dips^16^, safeguard generator synchronization with a power system^17^, and protect generators from fault-related consequences^18^. In^19^, most common FCLs were used at distribution systems. Before the FCLs can be widely adopted, two major technical concerns must be addressed^19^:

(I) Fault current restrictions may impact or even compromise the capacity of existing protection systems to detect faults, discriminate faults, and cooperate with other protection systems.
(II) Non-fault-related electrical transients, such as inrush currents of the transformer and large starting currents of the motor, can cause FCLs to malfunction.

### The impact of the FCL on conventional protection schemes

Firstly, the OCRs are mostly employed for protecting the outgoing and incoming feeders of the distribution systems. The fault current is inversely proportional to the relay operational time. In general, Eq. (1) could be used to estimate the operating time of the traditional OCR:

1 $$\:t=TMS\:\frac{A}{{\left(\frac{{I}_{sc}}{{I}_{p}}\right)}^{B}-1}$$

where, *Isc* is the measured short-circuit current, *Ip* is the selected pick-up current, and the values of *A* and *B* depend on the type of the OCR characteristic curve (i.e., normal inverse, very inverse, and extremely inverse)^20^.

According to CIGRE Working Group A3.10^21^, the FCL has many effects on the protection systems: relay setting, selectivity, time coordination between overcurrent relays, and harmony with downstream fuses. Group A3.16 continues to work on and develop recommendations regarding the impact of the FCL on protective devices, building on the prior work. For a given current-time curve, the FCL may delay an overcurrent relay trip operation (particularly for severe fault current limitation) since the fault current is lowered^22^. Coordination times between upstream and downstream relays (with distinct current-time curves) would also rise. This may increase stresses on the system components during a fault^23^. In^24^, the method examines the effects of the distorted current waveforms generated by specific types of fault current limiters on time-overcurrent protection relays in-depth. The experimental results were presented in paper^25^, which revealed that the distorted current and voltage waveforms caused by the FCL upset the protective devices. Power electronic switches were used to build and implement the experimental circuit that simulates the DG and protective devices in^26^. The paper^27^ used the model to conduct technical analysis and propose specifications for the Current Limiting Resistor/Reactor (CLR) that would be used to apply the FCL to a real Korean distribution power system. On the other hand, the FCL harms protection devices such as over-current relays. Blinding the protection, false sympathetic tripping, enclosure-fuse miss-coordination, a lapse of inter-fusing coordination, and failed autoclosing are the key protection difficulties related to the introduction of DERs and FCLs to the distribution network^28]– [29^. In the presence and absence of DGs, a comparison of OCR coordination for various fault locations was shown in^30^. The current ratio between the input current and the predetermined pick-up current (Ip) determines the OCR’s operation time. For protection coordination, the Time Dial (TD) can move the curve up or down. Both the parameters, Ip and TD can be manually modified in the general analog type of the OCR to suit protection coordination, but it isn’t an effective solution because of the frequent transfer of the current-time curve that will lead to overcurrent malfunction. It can be concluded from the numerous case studies that the FCL harms OCR coordination^9^. To avoid any malfunction of the protective relays, comprehensive investigations are necessary before implementing the FCL in the electrical systems^31^. In^4^, it was found that locating the FCL near the DG limits the current magnitude and reduces difficulties with existing OCR protection coordination. Short-circuit tests were used to investigate the impact of the FCL positioned into the feeder’s entry of the distributed power system on the operational features of the OCR^32^. Multiple factors were studied in^15^ to establish the ideal locations of the FCLs for protecting an electric power system with DGs, including the number of FCLs, fault current reduction, and total operating time of the relays. The numerical results demonstrated that the suggested approach can determine the FCL placement to reduce the fault current to be within the protective devices’ breaking capability while still fulfilling the CTI criteria of the relays. In^33^, the TD’s value and the OCR’s pickup current for protection coordination with the FCL were investigated, but these values had the risk of malfunctioning in normal operating conditions or establishing a standard.

In^25^, adaptive overcurrent relay settings are developed and tested in an IEEE 12 bus system under various operating situations. The use of the voltage component in the OCR was recommended in^34]– [35^ to minimize the trip delay induced by the FCL operation. However, the method is considered complicated because it relied on voltage measurements other than the current data, and not all feeders have voltage transformers. When using two-step overcurrent protection^36^, the same issues as with independent time OC relays might arise. The challenge is due to the limiting operation period of the FCL and the difficult selection of time steps once again^37^. In addition, the protection coordination investigations for the simulated power distribution system with the FCL were used to illustrate the approach of OCR-reclose protection coordination^20^. The resistance of the FCL should be adjusted to be within a certain range to retain the protection coordination, according to the analysis of input data^38^. When the FCL is used in a power system, the magnitude of the FCL’s impedance must be determined with a suitable value under the set rule of the protective overcurrent relay (OCR)^13^. The FCL will function and reduce the short-circuit current when a fault occurs. As a result, the OCR trip time will be delayed, especially in the case of severe fault current limiting. The operating time it takes for upstream and downstream relays to coordinate will be extended. During a relay malfunction, this scenario will raise the stress on the electrical equipment. Typically, conventional overcurrent relays will not work for much lower fault current, resulting in the creation of more setting groups for different scenarios as follows: (1) Without DG and without FCL, (2) With DG and without FCL, (3) Without DG and with FCL, and (4) With DG and with FCL.

**Fig. 1:**
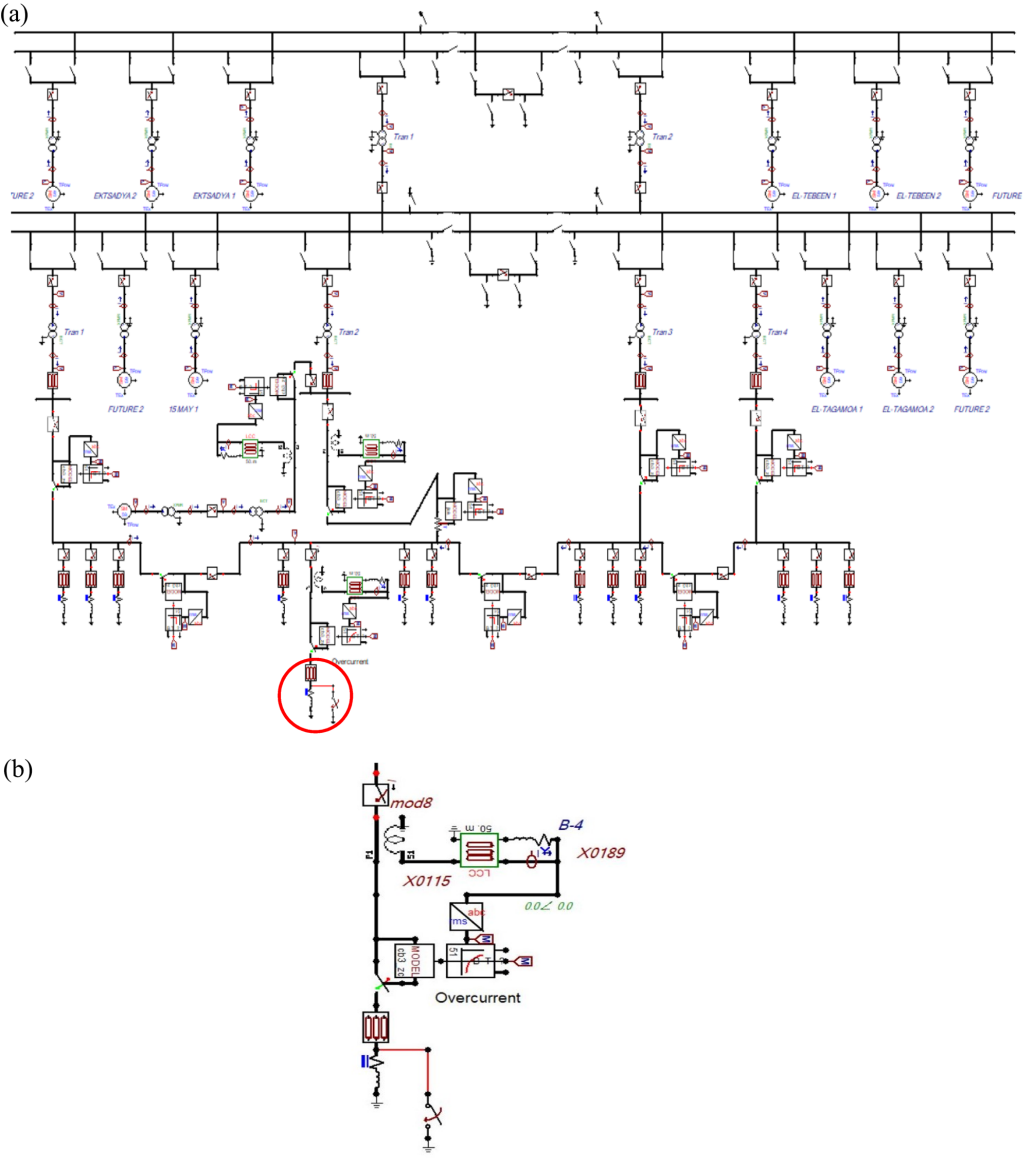
**a** The simulated power system model built on the ATP platform. **b** The relay and fault location (in red color) in the simulated power system model. **a**, **b** The simulated power system model under test.

This paper presents a new OCR algorithm to deal with the presence of the FCL and the DGs without malfunction of the protection system. In this study, the ATP program application is used to simulate a practical substation of 220/66/11 kV in Cairo city in Egypt country, as shown in Fig. 1.

### Main contributions

In this work, the main contributions to knowledge are enumerated as follows:

1. An adaptive overcurrent protection scheme is proposed to detect and select the faulty phase(s) using the Z-score function calculated for each phase current,
2. Current Mean Ratio (MR) is used to confirm the fault events and estimate the relay tripping time,
3. The approach presents only one type of protection tripping curve based on the Mean Ratio (MR) quantified for three-phase current signals, which is useful for both phase overcurrent and earth fault relays, and.
4. The methodology can control the relay tripping time using the current Mean Ratio (MR) that can be modified automatically, as well as the data set area that can be set to be one cycle or sub-cycle. This is accomplished according to the protection requirements and the prevailing condition of power systems.

The paper is ordered as follows: In “Configuration and modeling of the simulated power system”, a configuration and modeling for a typical simulated power system with a list of real parameters of its components is demonstrated. The strategy of fault detection and adaptive overcurrent protection is described in “Proposed method”. “ Results and discussion” introduces the quantitative findings of various case studies, along with analyzing the results. A comparison between the proposed solution and traditional OCRs is presented in “Critical comparison”. Finally, the conclusions are drawn in “Conclusions”.

##  Configuration and modeling of the simulated power system

Figure 1 shows the power system model designed using the ATP program application. This system is used to examine the performance of the proposed OCR under the effect of the FCLs with/without DGs. The influence of the FCLs on the sensitivity, reliability, and coordination of the overcurrent relays in the primary distribution system is the main topic of this research. The voltage power supply is 220 kV, which is further stepped down to 66 kV and 11 kV. The protection system has behavior that protects the power grid from serious or unstable circumstances. The OCR is introduced as one of the protection devices for maintaining power distribution systems. The operations of the OCR are categorized as instant time or delay time, with various interrupt characteristics depending on the amplitude of the pickup current of the OCR when the fault occurs. The trip signal is generated by the OCR and transferred to the circuit breakers (CBs) to isolate the fault zone from the remaining electrical system. In a 220 kV network, there are four feeders: (EL-TEBEEN 1, 2), (EKTSADYA 1, 2), and (Future 1, 2). In a 66 kV network, there are also four feeders: (EL-TAGAMOA 1, 2), (EKTSADYA 1, 2), and (Future 1, 2). A 3-phase substation transformer of 220/66 kV and 40 MVA has Y/Y0 windings connection with grounding Y and an 8% short-circuit impedance. A 3-phase substation transformer of 66/11 kV and 25 MVA has Δ-Y11 windings connection with grounding Y and an 11% short-circuit impedance. The power supplies of the electrical system are simulated by SM59 on the ATP platform. The ATP Line/Cable model is used to simulate copper cable feeders. The cable feeders are all the same XLPE type, 3-phase, single-core, 0.045 m radius, grounded from a single end, and have a laying depth of 0.922 m. The FCL is modeled by a non-linear resistor of type 91 and controller unit. The signal is produced from the controller to activate resistive impedance when the fault occurs. A conventional inverse OCR is simulated on the outgoing feeder that is supplied from incoming transformer 2 at busbar 2 of 11 kV. Table 1 includes the pickup current values of the incoming and outgoing feeders. The ATP program uses RLC3 to simulate each electrical load. The feeder load understudy has an active power of 10 MW and reactive power of 7 MVAR, with a power factor of 0.82. Appendix 1 presents the parameters’ data of the simulated power system components.

**Table 1:** Pickup values of incoming and outgoing feeders

Transformer 2 (incoming feeder)	Ioc (2*Ir)	2624 Amp
Outgoing feeder	Ioc (1.2*In)	600 Amp
	Is (6*In)	3600 Amp

##  Proposed method

### Fault detection

**Fig. 2:**
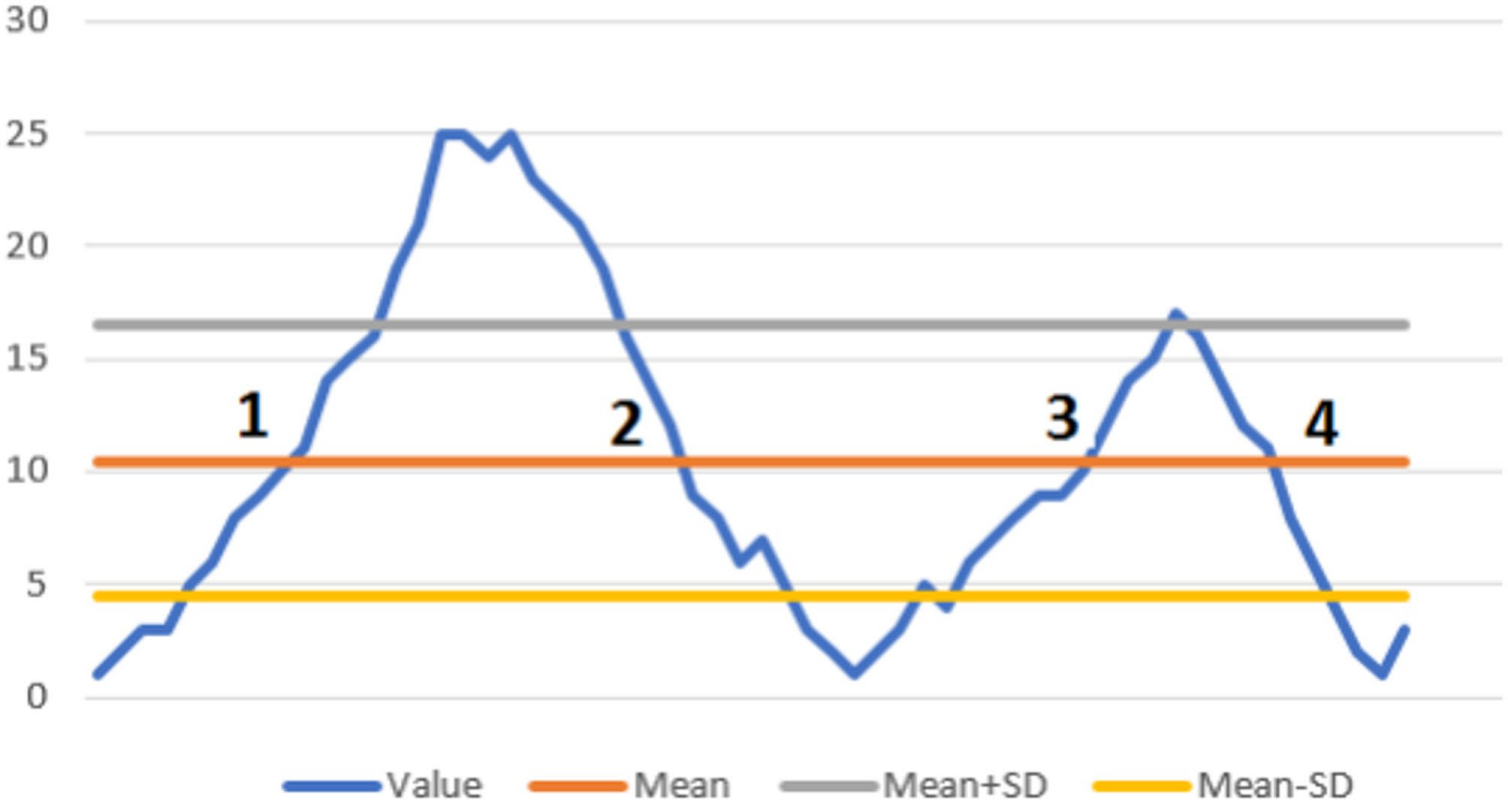
The Z-score algorithm applied: peak detection from the input data

The smoothed Z-score peak detection method analyzes the signal’s evolution using a changing mean and calculates standard deviations to establish a threshold around the signal. The algorithm detects the points that are outside this threshold as peaks. The technique is based on the idea of dispersion, where a data point is defined as a peak if the gap between it and the mean is a specified value of standard deviations^39^. Most peak detection methods include a lot of parameters, which makes them difficult to apply. The Z-score algorithm was selected since it has a few numbers of limitations. Three parameters are used in the algorithm: lag ($\:l$), influence ($\:{I}_{n}$), and threshold ($\:th$). The lag determines how adaptable the technique is (in terms of the long-term average of the data) as well as how smooth the data will be. The algorithm’s resilience will be improved by adding lag. If the lag is set to 5 samples, for example, the algorithm will react to the pattern once every 5 samples. The signal’s influence on the threshold is referred to as a consequence. Influence (0) denotes that the data has no impact on the threshold (that assumes a stable process). The influence should be adjusted between (0) and (1) if the signal is not stable. Signals have a 10% effect over the threshold if their influence is 0.1. The threshold is the value that determines whether a data point is a peak or not. It is the value of the standard deviation (std) from the moving mean. This parameter determines the algorithm’s sensitivity. Figure 2 illustrates what has been discussed thus far. The blue line represents the current time series, the orange line represents the mean, the gray line represents the “plus” line, and the yellow line represents the “minus” line.

It is detected by first measuring the mean power of each 30 log-spaced (denoted as $\:i$ = $\:{i}^{1}$, $\:{i}^{2}$, …, $\:{i}^{30}$) and then using a provided technique to find peaks in ($\:i$). The suggested method accepts $\:\left(i\right)$ as input and output is a vector y = [$\:{y}^{1}$, $\:{y}^{2}$, …, $\:{y}^{N}$], which is a series of “0” or “1”. In an anomalous scenario, zero signifies no peak identification, and one represents a positive or negative peak identified. Peaks are found in theory by creating a moving mean (µ) and a moving standard deviation (σ) from a smoothed signal $\:{(i}^{smooth})$. The algorithm then employs the notion that when the differences between the current value and the average value across a moving data window surpass a certain threshold, the current value is designated as a peak. The formulae for computing the Z-score based on adaptive peak identification are as follows:

2 $$\:{\mu\:}_{i}\:=\:{I}_{n}\:{x}_{i}+(1-\:{I}_{n})\:{\mu\:}_{i-1}$$

3 $$\:{\stackrel{-}{\mu\:}}_{i}=\:\frac{1}{l}\:\sum\:_{i}^{i+l}{\mu\:}_{i}$$

4 $$\:{\sigma\:}_{{\mu\:}_{i}}=\:\sqrt{\frac{\sum\:_{i}^{i+l}{\left({\mu\:}_{i}-{\stackrel{-}{\mu\:}}_{i}\right)}^{2}}{l-1}}$$

5 $$\:{z}_{i}=\:\frac{{x}_{i}-\:{\stackrel{-}{\mu\:}}_{i-1}}{{\sigma\:}_{{\mu\:}_{i-1}}}$$

6 $$\:{y}_{i}\:=\:\left\{\begin{array}{c}\pm\:1\:\:\:\:\:\:\:\:if\:\:\left|{z}_{i}\right|\:\ge\:th\\\:\:\\\:0\:\:\:\:\:\:\:\:\:\:\:\:if\:\:\left|{z}_{i}\right|\:\le\:th\end{array}\right.$$

where $\:\left(l\right)$ is the lag, $\:\left({I}_{n}\right)$ is the influence, $\:\left(th\right)$ is the threshold, $\:{(x}_{i})$ is the current signal, $\:{(\mu\:}_{i})$ is the signal after influence application, $\:\left({\stackrel{-}{\mu\:}}_{i}\right)$ is the mean, $\:\left({\sigma\:}_{{\mu\:}_{i}}\right)$ is the standard deviation, and $\:\left({y}_{i}\right)$ is the output signal.

In this study, the parameters are set as follows: lag = 30, threshold = 5 samples, and influence = 0. The following is a summary of the algorithm: Firstly, initialize $\:{i}^{smooth}$ using the first lag amount of data points in $\:\left(i\right)$, set µ = mean ($\:{i}^{smooth}$), and σ = std ($\:{i}^{smooth}$). For g = lag + 1 to N, make the following: If abs ($\:{i}^{g}$ − µ) > threshold × σ, the algorithm indicated a “1” (positive) for abnormal conditions. Otherwise, if abs ($\:{i}^{g}$ − µ) < threshold × σ, it is considered a normal case. The condition for the smoothed Z-score method is shown in the following equation:

7 $$\: \left|\:i\:-avg\left(last\:cycle\right)\right| >\:th*std\left(last\:cycle\right)$$.

**Fig. 3:**
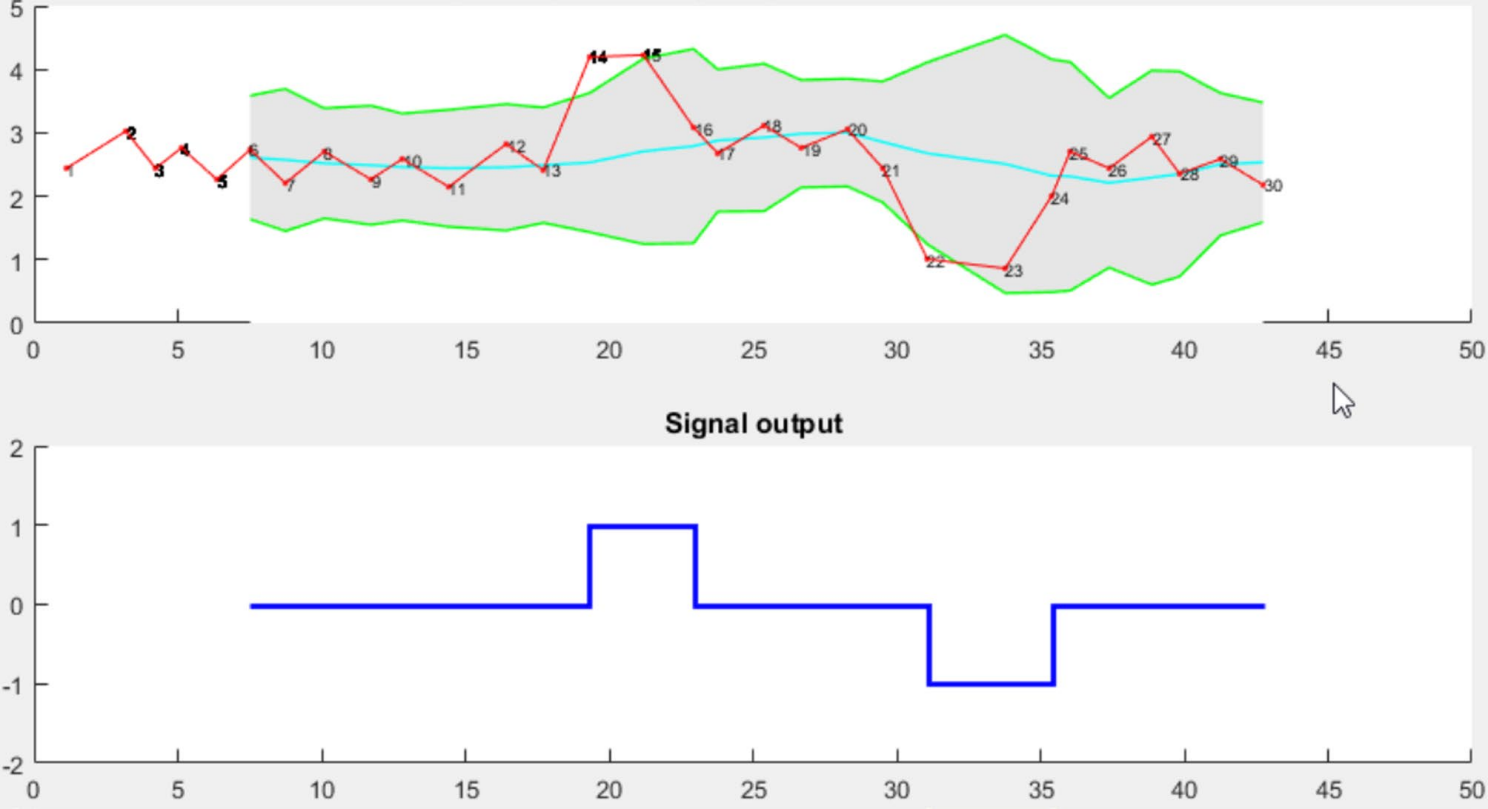
Output signal with Z-score algorithm

Finally, the signal results are represented in Fig. 3 using a simple line graph to illustrate the output signal that specifies the peak values in the abnormal cases.

The developed algorithm can address this issue without depending on the current amplitude that may be limited by the FCL. Firstly, the three-phase currents are measured. After that, for each cycle, calculate the average and standard deviation. The protection relay is active according to Eq. (6). If that condition is verified, the OCR operation will start to detect fault instances. Appendix 2 illustrates the input quantities for the protection algorithm.

### Adaptive OCR algorithm

**Fig. 4:**
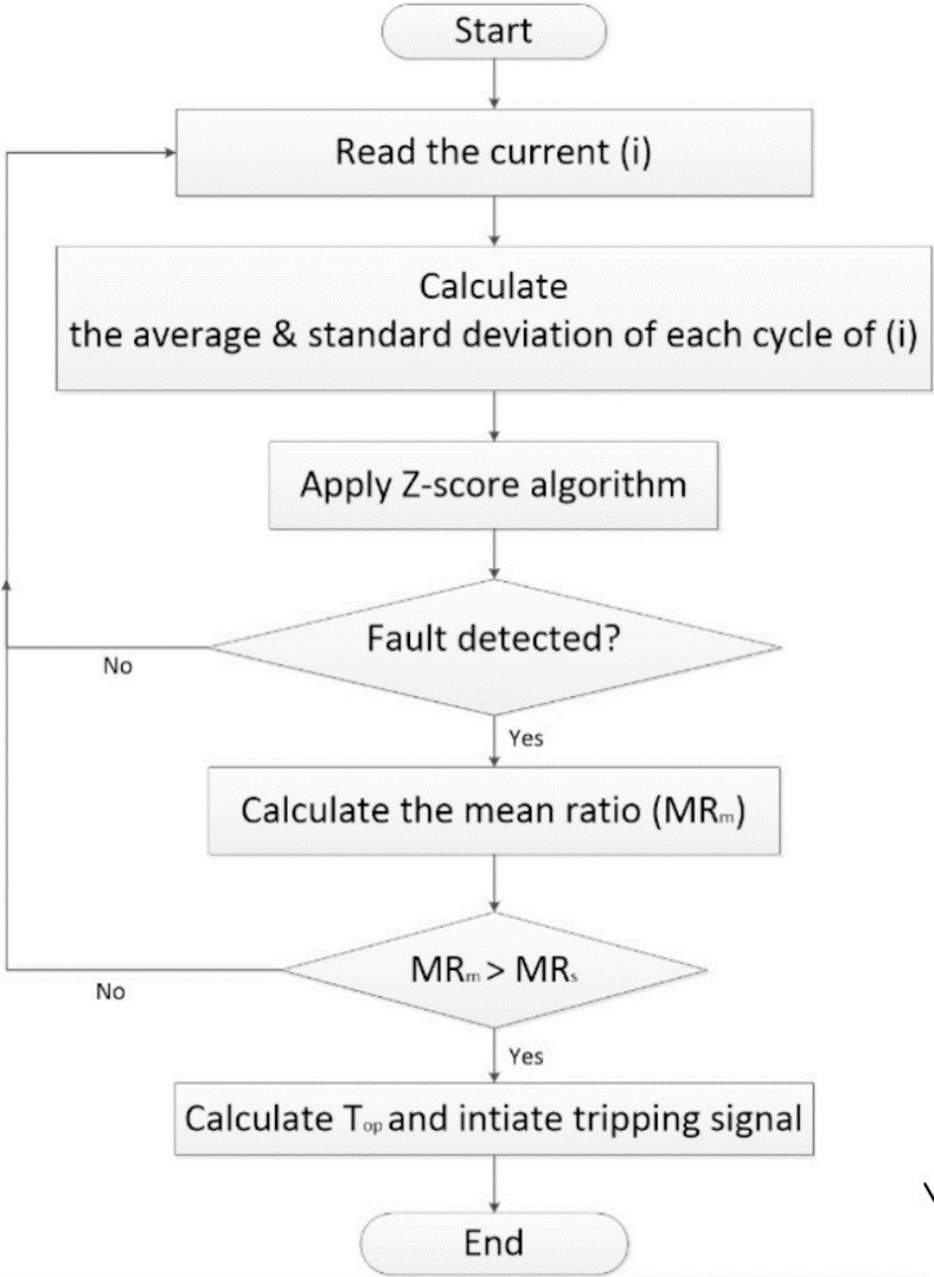
Flowchart of the proposed protection algorithm

To make the OCRs function efficiently in any system condition, they should be provided with an adaptive setting. The flowchart of the proposed algorithm is illustrated in Fig. 4. In this section, the intelligent protection algorithm will be described, which is proposed for electrical distribution networks with the presence of Distributed Generators (DGs). This algorithm presents a superior solution for the inverse OCRs. This contradiction may happen when a fault occurs and the FCL enters service to limit fault current. The limited current can affect the effectiveness of the protection OCRs, leading to a delay in the tripping time.

After fault detection, the current Mean Ratio (MR) is applied to calculate the tripping time and clear the fault event from the power network. This method is effective for tripping circuit breakers without any time delay. It operates automatically without any need to set a pickup current value. It can estimate the operating time based on the Mean Ratio (MR) of the phase current signal, which originates after the relay initiation using the Z-score algorithm. In this case, the MR will be calculated automatically by dividing the average current for a given cycle by the average current for the previous cycle. Equation (8) presents the formula for calculating the current Mean Ratio (MR). The algorithm assures the fault presence when the measured value of the mean ratio $\:{(MR}_{m})$ is greater than the setting value of the mean ratio$\:{(MR}_{s})$. Inequality (9) can be applied to perform this function. Equation (10) expresses the mathematical formula for calculating the tripping time (*t*) of the adaptive OCR.

- $\:{\:MR}_{s}$: The setting value of the current mean ratio, which is adapted by calculating the mean ratio at the previous cycle.
- $\:{\:MR}_{m}$: The measured current mean ratio at the present cycle.
- $\:{I}_{avg}{\:)}_{n}$: The average current (in Amp) for the present cycle.
- $\:{I}_{avg}\:{)}_{n-Nc}$: The average current (in Amp) for the previous cycle.
- t: The relay operating time (in Sec).

8 $$\:MR\:=\frac{{I}_{avg}{\:)}_{n}}{{I}_{avg}\:{)}_{n-Nc}}$$

9 $$\:{MR}_{m}\:\:>\:\:{MR}_{s}$$

10 $$\:t=TMS\:\frac{A}{[{\left(\frac{{I}_{avg}{\:)}_{n}}{{I}_{avg}\:{)}_{n-Nc}}\right)}^{B}-1]}$$

### Sensitivity analysis of the AOCR

The sensitivity analysis is the study of whether the secondary relay is sensitive enough to respond to a low fault current situated at the far terminal of its primary relay protection zone. The sensitivity analysis is an important aspect of the relays’ coordination study. Coordination pairs whose secondary relays do not verify the requirement of sensitivity will cause long operating times. The sensitivity of the developed AOCR relies on the following criteria:

11 $$Sensitivity \propto MR=\frac{{{{\left. {{I_{avg}}} \right)}_n}}}{{{{\left. {{I_{avg}}} \right)}_{n - {N_c}}}}}$$.

The AOCR algorithm will be more sensitive when the setting values (MR) are greater. As the load current or the short circuit current increases, the mean ratio (MR) values will rise. This will result in an increase in the protection’s sensitivity. Thus, automatic online coordination will be accomplished.

Also, the AOCR algorithm will become more sensitive when the data window set is lower. In this method, the data window set can be used to control the protection’s sensitivity.

12 $$\:\text{S}\text{e}\text{n}\text{s}\text{i}\text{t}\text{i}\text{v}\text{i}\text{t}\text{y}\:{\upalpha\:}\:\left|\frac{1}{\text{D}\text{a}\text{t}\text{a}\:\text{w}\text{i}\text{n}\text{d}\text{o}\text{w}\:\text{s}\text{e}\text{t}}\right|$$

The data window set and the settings (MR) of the protection function have the subsequent influences on the protection characteristics:

1. In the instance of low fault current, the protection’s sensitivity can be enhanced by decreasing the data window set or increasing the MR settings of the protection function. This is like the reduction in the pickup current for the existing overcurrent relays.
2. In the instance of overload current, the protection’s security can be elevated by increasing the data window set or decreasing the MR settings of the protection function. This is similar to the increase in the pickup current for the existing overcurrent relays.

As a result, a compromise in the relay settings is still required to ensure coordination between the security and sensitivity of the AOCR algorithm.

##  Results and discussion

The effectiveness of the proposed methodology is successfully tested for various cases in ATPDraw without being affected by the presence or absence of FCLs and/or DGs. In this study, the following cases are presented with different fault locations on the cable feeder:

1. Different types of faults.
2. Different fault inception times.
3. Different fault impedances.
4. Operating load changes with/without faults.

All tests are achieved using different fault locations that range from 100 m to 5 km of the feeder cable of Transformer 2. This study aims to validate the proposal effectiveness, and differentiate between the conventional OCR (that is built by the ATP application) and the adaptive OCR (that is simulated by ATP platform and its algorithm is processed using the MATLAB M-file software).

### Different fault types

#### Case 1: single line-to-ground fault at t_f_ = 0.06 s

**Fig. 5:**
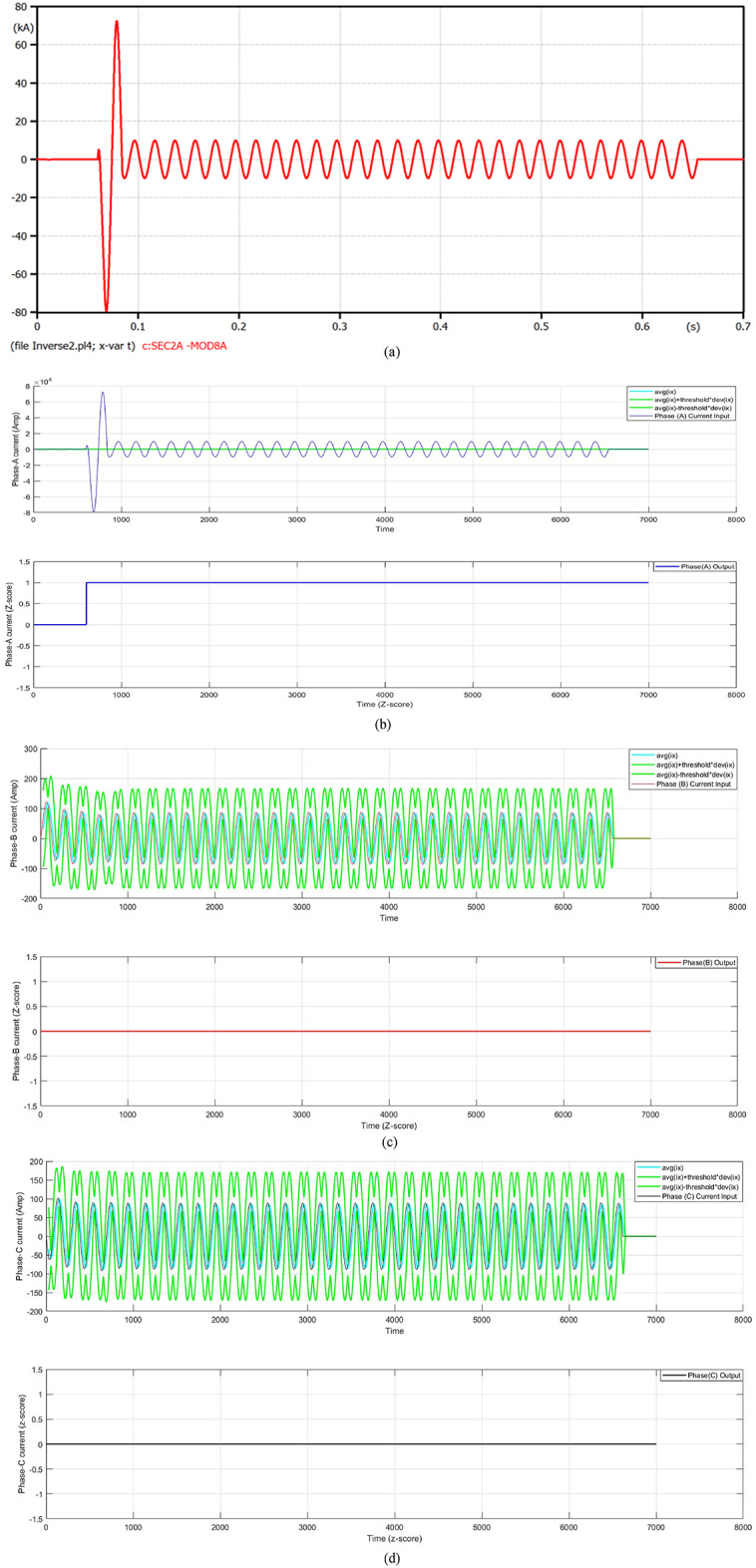
**a**–**d** Algorithm results in the case of a single line-to-ground fault at 0.06 s

In this case, a single line-to-ground fault (SLGF) is situated on the distribution feeder cable of Transformer 2 at the time instant 0.06 s. Figure 5 presents a fault current with a fault distance of 100 m. The fault current increases to 72 kA after one cycle of the fault inception time, then the FCL limits the fault current. At that moment, the fault current is reduced to 9.732 kA. In this instant, the Conventional Overcurrent Relay (COCR) operating time (OPT) is increased from 0.3476 s to 0.5754 s. Whereas, the proposed Adaptive Overcurrent Relay (AOCR) operating time is not affected with or without the FCL, where the OPT is equal to 0.34424 s. Table 2 shows the values of the tripping time for the conventional and adaptive OCRs for different locations (on the feeder cable) of single line-to-ground faults with *R*_*f*_ = 0.0 Ω at *t*_*f*_ = 0.06 s.

When using the conventional OCR at 100 m, the OPT is close to 0.3887 s in the case of without DG and without FCL, but it varies to 0.5847 s in the case of without DG and with FCL. Whereas, the OPT value of the AOCR remains the same in both cases, which equals 0.35550 s. But the OPT value of the COCR at 100 m is roughly 0.3476 s in the case of with DG and without FCL, but it changes to 0.5754 s in the case of with DG and with FCL. Nevertheless, the OPT value of the AOCR is constant for both cases, which is equal to 0.34424 s. For all scenarios of the SLGFs, the quantitative results show that the AOCR is faster than the COCR, as shown in Table 2.

Table 2 presents the operating times (OPT) at diverse distances of the cable when the running load of the protected feeder is 100% of the base load (Z_L_ = j120.26 + j26.99 Ω) and a single line-to-ground fault (SLGF) condition with *R*_*f*_ = 0.0 Ω at *t*_*f*_ = 0.06 s. The operating times of the conventional and adaptive OCRs are estimated with/without FCLs and DGS.

**Table 2:** Operating time for single line-to-ground fault with *R*_*f*_ = 0.0 Ω at *t*_*f*_ = 0.06 s

Distance	Operating time (OPT) Single line-to-ground fault with Rf = 0.0 Ω at t_f_ = 0.06 s Z_L_ = j120.26 + j26.99 Ω							
	Conventional OCR				Adaptive OCR			
	Without FCL Without DGSM	Without FCL With DGSM	With FCL Without DGSM	With FCL With DGSM	Without FCL Without DGSM	Without FCL With DGSM	With FCL Without DGSM	With FCL With DGSM
100 m	0.3887	0.3476	0.5847	0.5754	**0.35550**	**0.34424**	**0.35550**	**0.34424**
300 m	0.4775	0.4252	0.6252	0.6241	0.40643	0.40021	0.40643	0.40021
500 m	0.5366	0.5180	0.6434	0.6419	0.44169	0.43709	0.44169	0.43709
700 m	0.5865	0.5756	0.6633	0.6629	0.46987	0.46611	0.46987	0.46611
900 m	0.5898	0.5892	0.6770	0.6740	0.49392	0.49066	0.49392	0.49066
1100 m	0.6152	0.6150	0.6933	0.6912	0.51520	0.51230	0.51520	0.51230
1300 m	0.6458	0.6345	0.7039	0.7021	0.53450	0.53186	0.53450	0.53186
1500 m	0.6557	0.6540	0.7233	0.7224	0.55229	0.54985	0.55229	0.54985
1700 m	0.6755	0.6742	0.7340	0.7340	0.56887	0.56659	0.56887	0.56659
1900 m	0.6884	0.6846	0.7442	0.7440	0.58450	0.58234	0.58450	0.58234
2100 m	**0.7044**	**0.7013**	**0.7044**	**0.7013**	0.59931	0.59725	0.59931	0.59725
2500 m	0.7345	0.7239	0.7345	0.7239	0.62695	0.62508	0.62695	0.62508
3000 m	0.7645	0.7631	0.7645	0.7631	0.65869	0.65696	0.65869	0.65696
3500 m	0.7939	0.7929	0.7939	0.7929	0.68810	0.68645	0.68810	0.68645
4000 m	0.8246	0.8142	0.8246	0.8142	0.71559	0.71413	0.71559	0.71413
4500 m	0.8442	0.8437	0.8442	0.8437	0.74170	0.74025	0.74170	0.74025
5000 m	0.8752	0.8741	0.8752	0.8741	0.76663	0.76525	0.76663	0.76525

#### Case 2: double line fault at t_f_ = 0.06 s

**Fig. 6:**
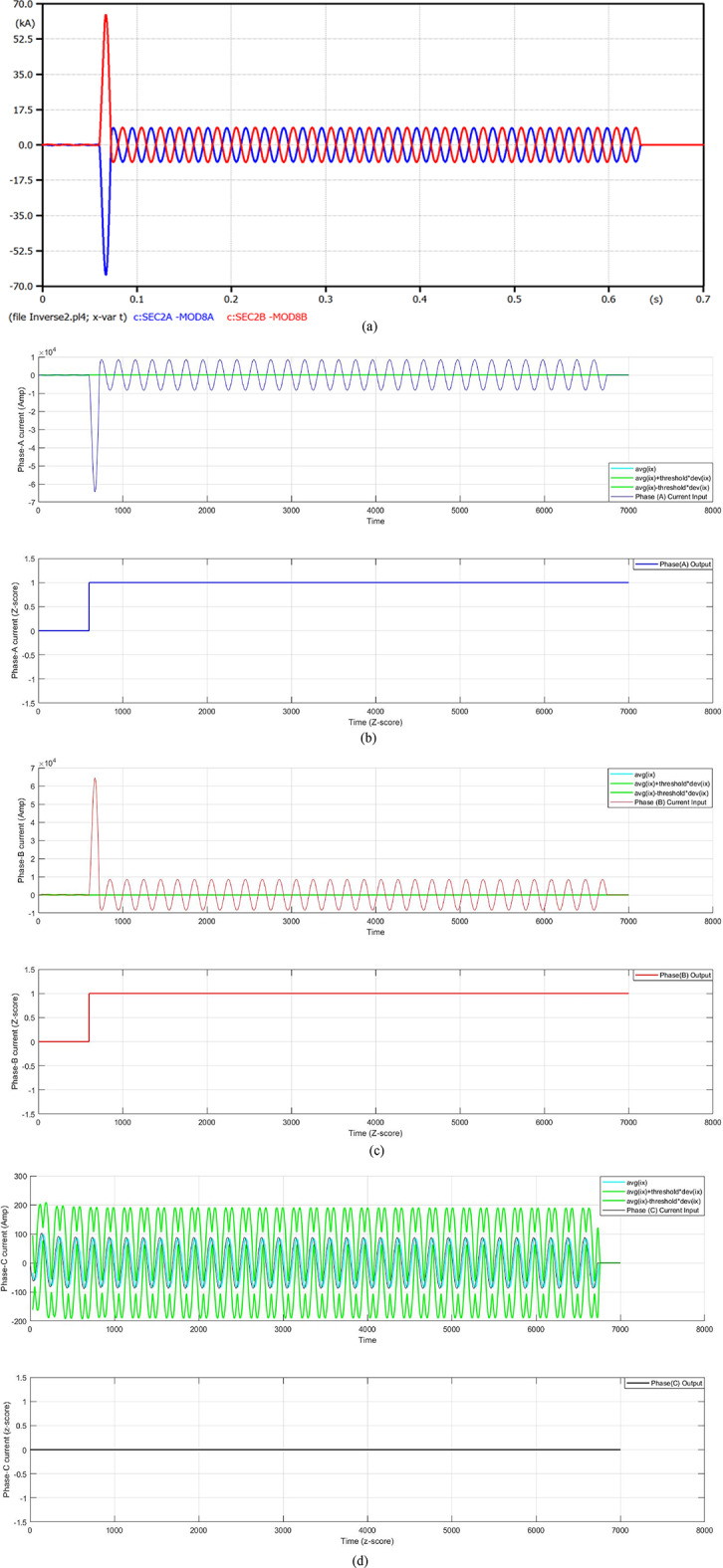
**a**–**d** Algorithm results in the case of a double line fault at 0.06 s

In this case, a double line fault (DLF) is located on the distribution feeder of the transformer 2 at 0.06 s. Figure 6 presents a fault current in the case of a fault distance equal to 100 m. After one cycle of the fault starting, the fault current rises to 64.35 kA, and the fault current is limited by the FCL. At that moment, the fault current decreases to 8.390 kA, but the COCR operating time (OPT) is extended from 0.3578 s to 0.5725 s. In this instance, the proposed AOCR operating time is not affected by the FCL entrance, where the OPT is equal to 0.35090 s. Table 3 includes the values of the tripping time for the conventional and adaptive OCRs for different locations (on the feeder cable) of double line faults, with *R*_*f*_ = 0.0 Ω at *t*_*f*_ = 0.06 s.

When using the conventional OCR at 100 m, the OPT is nearly 0.3985 s in the situation of without DG and without FCL, but it varies to 0.6249 s in the case of without DG and with FCL. Therewith, the OPT value of the adaptive OCR is fixed for both cases, which equals 0.36130 s. But the OPT of the conventional OCR is about 0.3578 s in the case of with DG and without FCL, but it varies to 0.5725 s in the case of with DG and with FCL. Notwithstanding, the OPT value of adaptive OCR remains the same in both instances, which is equal to 0.35090 s. It is seen that the AOCR is quicker than the COCR for all scenarios of the DLFs, as illustrated in Table 3.

Table 3 includes the operating times (OPT) at disparate distances of the cable when the running load of the protected feeder is 100% of the base load (Z_L_ = j120.26 + j26.99 Ω) and a double line fault (DLF) condition with *R*_*f*_ = 0.0 Ω at *t*_*f*_ = 0.06 s. The operating times of the conventional and adaptive OCRs are calculated with/without FCLs and DGS.

**Table 3:** Operating time for double line fault with *R*_*f*_ = 0.0 Ω at *t*_*f*_ = 0.06 s

Distance	Operating time (OPT) Double line fault with Rf = 0.0 Ω at t_f_ = 0.06 s Z_L_ = j120.26 + j26.99 Ω							
	Conventional OCR				Adaptive OCR			
	Without FCL Without DGSM	Without FCL With DGSM	With FCL Without DGSM	With FCL With DGSM	Without FCL Without DGSM	Without FCL With DGSM	With FCL Without DGSM	With FCL With DGSM
100 m	0.3985	0.3578	0.6249	0.5725	**0.36130**	**0.35090**	**0.36130**	**0.35090**
300 m	0.4876	0.4655	0.6460	0.6043	0.41220	0.40646	0.41220	0.40646
500 m	0.5772	0.4849	0.6655	0.6221	0.44817	0.44396	0.44817	0.44396
700 m	0.5240	0.5183	0.6850	0.6416	0.47713	0.47370	0.47713	0.47370
900 m	0.6054	0.5740	0.7027	0.6543	0.50197	0.49899	0.50197	0.49899
1100 m	0.6350	0.5940	0.7121	0.6730	0.52401	0.52138	0.52401	0.52138
1300 m	0.6553	0.6152	0.7323	0.6918	0.54407	0.54166	0.54407	0.54166
1500 m	0.6746	0.6345	0.7472	0.7030	0.56262	0.56038	0.56262	0.56038
1700 m	0.6940	0.6535	0.7670	0.7158	0.57996	0.57787	0.57996	0.57787
1800 m	**0.7053**	**0.6638**	**0.7053**	**0.7238**	0.58827	0.58624	0.58827	0.58624
1900 m	0.7143	0.6652	0.7043	0.6652	0.59633	0.59435	0.59633	0.59435
2000 m	0.7238	0.6728	0.7238	0.6728	0.60419	0.60229	0.60419	0.60229
2500 m	0.7568	0.7153	0.7568	0.7153	0.64104	0.63933	0.64104	0.63933
3000 m	0.7934	0.7472	0.7934	0.7472	0.67474	0.67313	0.67474	0.67313
3500 m	0.8238	0.7802	0.8238	0.7802	0.70607	0.70463	0.70607	0.70463
4000 m	0.8544	0.8099	0.8544	0.8099	0.73558	0.73427	0.73558	0.73427
4500 m	0.8820	0.8375	0.8820	0.8375	0.76377	0.76242	0.76377	0.76242
5000 m	0.9073	0.8645	0.9073	0.8645	0.79087	0.78953	0.79087	0.78953

#### Case 3: double line-to-ground fault at t_f_ = 0.06 s

**Fig. 7:**
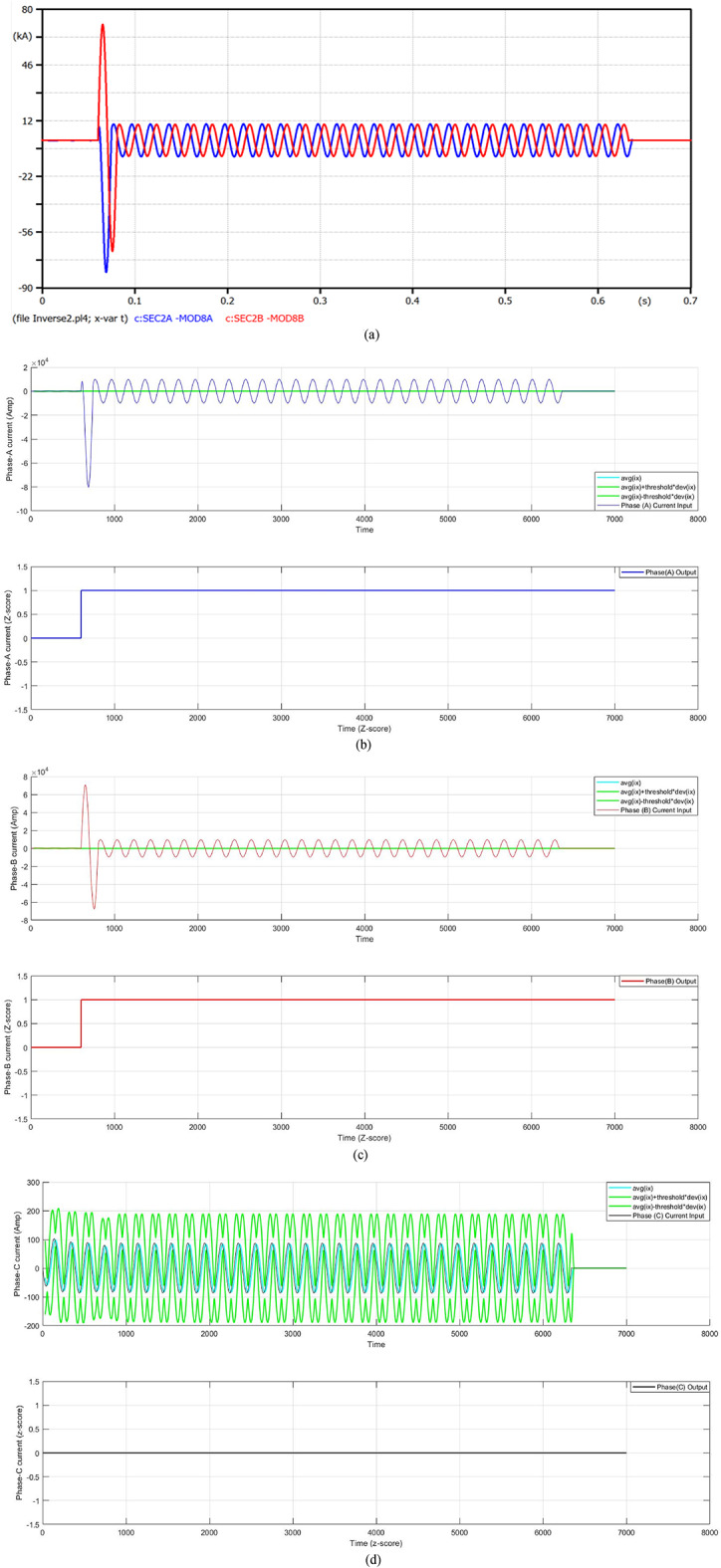
**a**–**d** Algorithm results in the case of a double line-to-ground fault at 0.06 s

In this case, a double line-to-ground fault (DLGF) originates on the distribution feeder of transformer 2 at the time 0.06 s. Figure 7 presents the fault current in the case of a fault distance that is equal to 100 m. The fault current surges to 80.27 kA after one cycle of the fault onset, then the FCL restricts the fault current. At that moment, the fault current is minimized to 9.805 kA, but the COCR operating time (OPT) is elevated from 0.3703 s to 0.5655 s. In this case, the proposed AOCR operating time is the same value with/without the FCL, where the OPT is equal to 0.34506 s. Table 4 illustrates the values of the operating time for the conventional and adaptive OCRs for different locations (on the feeder cable) of double line-to-ground faults with *R*_*f*_ = 0.0 Ω at *t*_*f*_ = 0.06 s.

When using the conventional OCR at 100 m, the OPT is approximately 0.3809 s in the case of without DG and without FCL, but it changes to 0.5663 s in the case of without DG and with FCL. When using the adaptive OCR, the OPT remains the same value for both cases, which is about 0.35592 s. Moreover, when using the conventional OCR at 100 m, the OPT is nearly 0.3703 s in the case of with DG and without FCL, but it changes to 0.5655 s in the case of with DG and with FCL. When using the adaptive OCR, the OPT value is fixed for both cases, which equals 0.34506 s. From the quantitative findings in Table 4, it is obvious that the AOCR is faster than the COCR for all scenarios of the DLGFs.

Table 4 illustrates the operating times (OPT) at different distances of the cable when the running load of the protected feeder is 100% of the base load (Z_L_ = j120.26 + j26.99 Ω) and a double line-to-ground fault (DLGF) condition with *R*_*f*_ = 0.0 Ω at *t*_*f*_ = 0.06 s. The operating times of the conventional and adaptive OCRs are computed with/without FCLs and DGS.

**Table 4:** Operating time for double line-to-ground fault with *R*_*f*_ = 0.0 Ω at *t*_*f*_ = 0.06

Distance	Operating time (OPT) Double line-to-ground fault with Rf = 0.0 Ω at t_f_ = 0.06 s Z_L_ = j120.26 + j26.99 Ω							
	Conventional OCR				Adaptive OCR			
	Without FCL Without DGSM	Without FCL With DGSM	With FCL Without DGSM	With FCL With DGSM	Without FCL Without DGSM	Without FCL With DGSM	With FCL Without DGSM	With FCL With DGSM
100 m	0.3809	0.3703	0.5663	0.5655	**0.35592**	**0.34506**	**0.35592**	**0.34506**
300 m	0.4387	0.4362	0.6363	0.6352	0.40620	0.40026	0.40620	0.40026
500 m	0.5493	0.5380	0.6460	0.6447	0.44137	0.43702	0.44137	0.43702
700 m	0.5682	0.5570	0.6655	0.6642	0.46955	0.46601	0.46955	0.46601
900 m	0.5890	0.5768	0.6757	0.6744	0.49360	0.49055	0.49360	0.49055
1100 m	0.6162	0.6058	0.6951	0.6942	0.51490	0.51219	0.51490	0.51219
1300 m	0.6359	0.6349	0.7048	0.7039	0.53420	0.53174	0.53420	0.53174
1500 m	0.6557	0.6548	0.7251	0.7242	0.55200	0.54973	0.55200	0.54973
1700 m	0.6755	0.6751	0.7355	0.7340	0.56589	0.56480	0.56589	0.56480
1900 m	0.6855	0.6844	0.7442	0.7437	0.58420	0.58223	0.58420	0.58223
2100 m	**0.7053**	**0.7039**	**0.7053**	**0.7039**	0.59903	0.59716	0.59903	0.59716
2500 m	0.7345	0.7251	0.7345	0.7251	0.62671	0.62496	0.62671	0.62496
3000 m	0.7650	0.7639	0.7650	0.7639	0.65848	0.65686	0.65848	0.65686
3500 m	0.7849	0.7841	0.7849	0.7841	0.68784	0.68639	0.68784	0.68639
4000 m	0.8243	0.8145	0.8243	0.8145	0.71542	0.71397	0.71542	0.71397
4500 m	0.8448	0.8437	0.8448	0.8437	0.74151	0.74015	0.74151	0.74015
5000 m	0.8741	0.8724	0.8741	0.8724	0.76640	0.76514	0.76640	0.76514

#### Case 4: three lines-to-ground fault at t_f_ = 0.06 s

**Fig. 8:**
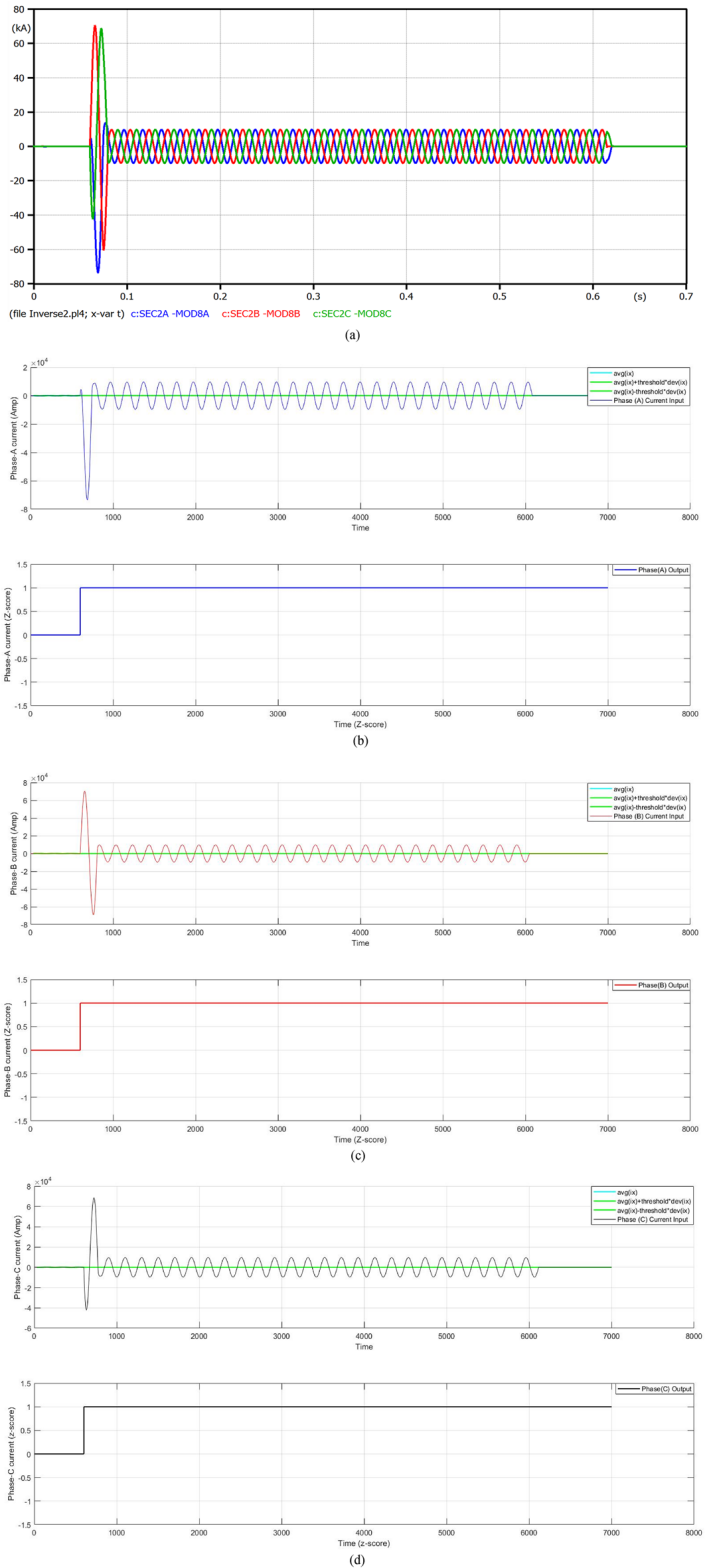
**a**–**d** Algorithm results in the case of a three-line fault at 0.06 s

In this case, a three line-to-ground fault (3LGFs) occurs on the distribution feeder at transformer 2 at the time 0.06 s. Figure 8 presents a fault current in the case of the fault distance that is equal to 100 m. The fault current magnitude grows to 73.44 kA after one cycle of the fault initiation time, then the FCL suppresses the fault current. At that moment, the fault current is reduced to 9.552 kA, but the COCR operating time (OPT) is expanded from 0.3731 s to 0.5477 s. In this illustration, the proposed AOCR operating time is not affected by the FCL entrance (i.e., it is steady whether with or without the FCL), where the OPT is 0.34982 s. Table 5 demonstrates the values of the operating time for the conventional and adaptive OCRs for different locations (on the feeder cable) of three line-to-ground faults with *R*_*f*_ = 0.0 Ω at *t*_*f*_ = 0.06 s.

When using the conventional OCR at 100 m, the OPT is close to 0.4039 s in the case of without DG and without FCL, but it varies to 0.5803 s in the case of without DG and with FCL. When using the adaptive OCR, the OPT value remains the same for both cases, where their values equals 0.36034 s. In addition, when using the conventional OCR at 100 m, the OPT is about 0.3731 s in the case of with DG and without FCL, but it becomes 0.5477 s in the case of with DG and with FCL. When using the adaptive OCR, the OPT remains the same value for both cases, where it is equal to 0.34982 s. From the quantitative results in Table 5, it is observed that the AOCR speed is superior to the COCR speed for all scenarios of the 3LGFs.

Table 5 presents the operating times (OPT) at various distances of the cable when the running load of the protected feeder is 100% of the base load (Z_L_ = j120.26 + j26.99 Ω) and a three-line fault (3LF) condition with *R*_*f*_ = 0.0 Ω at *t*_*f*_ = 0.06 s. The operating times of the conventional and adaptive OCRs are calculated with/without FCLs and DGS.

**Table 5:** Operating time for three-line fault with *R*_*f*_ = 0.0 Ω at *t*_*f*_ = 0.06 s

Distance	Operating time (OPT) Three-line fault with Rf = 0.0 Ω at t_f_ = 0.06 s Z_L_ = j120.26 + j26.99 Ω							
	Conventional OCR				Adaptive OCR			
	Without FCL Without DGSM	Without FCL With DGSM	With FCL Without DGSM	With FCL With DGSM	Without FCL Without DGSM	Without FCL With DGSM	With FCL Without DGSM	With FCL With DGSM
100 m	0.4039	0.3731	0.5803	0.5477	**0.36034**	**0.34982**	**0.36034**	**0.34982**
300 m	0.4829	0.4402	0.6291	0.6279	0.40917	0.40315	0.40917	0.40315
500 m	0.5415	0.5402	0.6483	0.6469	0.44369	0.43923	0.44369	0.43923
700 m	0.5623	0.5613	0.6678	0.6664	0.47150	0.46784	0.47150	0.46784
900 m	0.5870	0.5860	0.6780	0.6762	0.49532	0.49215	0.49532	0.49215
1100 m	0.6183	0.6176	0.6967	0.6956	0.51645	0.51363	0.51645	0.51363
1300 m	0.6381	0.6372	0.7075	0.7057	0.53563	0.53305	0.53563	0.53305
1500 m	0.6585	0.6566	0.7260	0.7251	0.55332	0.55095	0.55332	0.55095
1700 m	0.6782	0.6773	0.7361	0.7350	0.56984	0.56761	0.56984	0.56761
1900 m	0.6898	0.6887	0.7472	0.7457	0.58539	0.58332	0.58539	0.58332
2050 m	**0.7075**	**0.6965**	**0.7075**	**0.6965**	0.59655	0.59453	0.59655	0.59453
2500 m	0.7361	0.7292	0.7361	0.7292	0.62776	0.62591	0.62776	0.62591
3000 m	0.7658	0.7650	0.7658	0.7650	0.65943	0.65774	0.65943	0.65774
3500 m	0.7966	0.7949	0.7966	0.7949	0.68877	0.68718	0.68877	0.68718
4000 m	0.8253	0.8156	0.8253	0.8156	0.71624	0.71477	0.71624	0.71477
4500 m	0.8461	0.8444	0.8461	0.8444	0.74229	0.74092	0.74229	0.74092
5000 m	0.8752	0.8660	0.8752	0.8660	0.76720	0.76582	0.76720	0.76582

### Different inception times

#### Case 5: single line-to-ground fault at t_f_ = 0.063 s

**Fig. 9:**
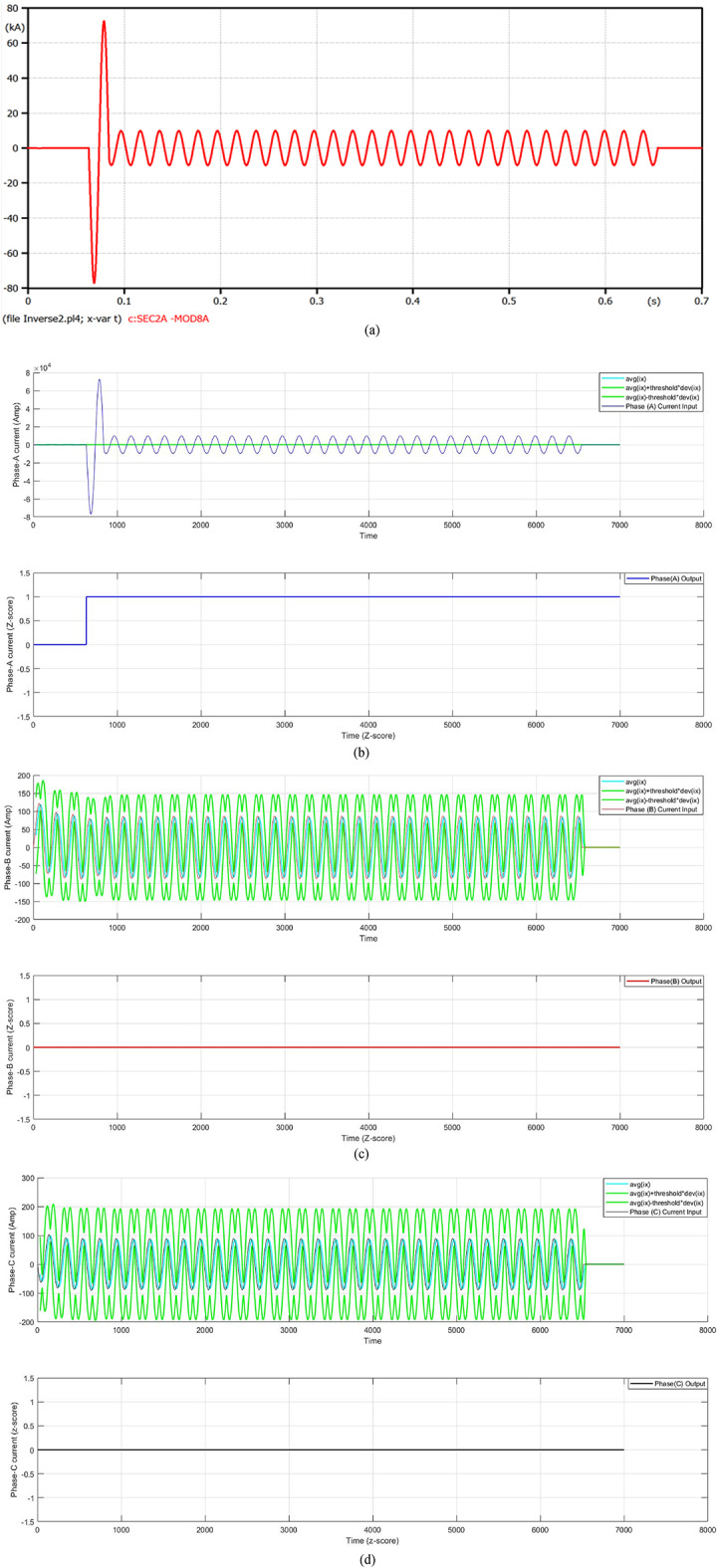
**a**–**d** Algorithm results in the case of a single line-to-ground fault at 0.063 s

In this case, a single line-to-ground fault (DLGF) is extent on the distribution feeder at the transformer 2 at the time instant 0.063 s. Figure 9 presents a fault current with a fault distance equal to 100 m. The fault current ascends to 77.06 kA after one cycle of the fault commencement, then the FCL reduces the fault current. At that moment, the fault current is decreased to 9.736 kA, but the COCR operating time (OPT) changes from 0.3565 s to 0.5946 s. In this case study, the proposed AOCR operating time is not affected by the FCL entrance (i.e., it is constant with or without the FCL), where the OPT equals to 0.34744 s. Table 6 lists the values of the operating time for the conventional and adaptive OCRs for different locations (on the feeder cable) of single line-to-ground faults with *R*_*f*_ = 0.0 Ω at *t*_*f*_ = 0.063 s.

When using the conventional OCR at 100 m, the OPT is equal to 0.3770 s in the case of without DG and without FCL, but it varies to 0.6048 s in the case of without DG and with FCL. When using an adaptive OCR, the OPT value is fixed for both cases, which is roughly 0.35895 s. Furthermore, when using the conventional OCR at 100 m, the OPT is nearly 0.3565 s in the case of with DG and without FCL, but it is adapted to 0.5946 s in the case of with DG and with FCL. When using the adaptive OCR, the OPT remains the same value in both cases, where it is equal to 0.34744 s. From the quantitative findings in Table 6, it is clear that the AOCR is faster than the COCR for all scenarios of the DLGFs.

Table 6 includes the operating times (OPT) at different distances of the cable when the running load of the protected feeder is 100% of the base load (Z_L_ = j120.26 + j26.99 Ω) and a single line-to-ground fault (SLGF) condition with *R*_*f*_ = 0.0 Ω at *t*_*f*_ = 0.063 s. The operating times of the conventional and adaptive OCRs are estimated with/without FCLs and DGS.

**Table 6:** Operating time for single line-to-ground fault with *R*_*f*_ = 0.0 Ω at *t*_*f*_ = 0.063 s

Distance	Operating time (OPT) Single line-to-ground fault with Rf = 0.0 Ω at t_f_ = 0.063 s Z_L_ = j120.26 + j26.99 Ω							
	Conventional OCR				Adaptive OCR			
	Without FCL Without DGSM	Without FCL With DGSM	With FCL Without DGSM	With FCL With DGSM	Without FCL Without DGSM	Without FCL With DGSM	With FCL Without DGSM	With FCL With DGSM
100 m	0.3770	0.3565	0.6048	0.5946	**0.35895**	**0.34744**	**0.35895**	**0.34744**
300 m	0.4850	0.4151	0.6249	0.6240	0.41064	0.40426	0.41064	0.40426
500 m	0.5250	0.4944	0.6452	0.6443	0.44654	0.44182	0.44654	0.44182
700 m	0.5840	0.5747	0.6638	0.6629	0.47529	0.47143	0.47529	0.47143
900 m	0.5937	0.5850	0.6833	0.6735	0.49985	0.49651	0.49985	0.49651
1100 m	0.6145	0.6139	0.6940	0.6933	0.52161	0.51862	0.52161	0.51862
1300 m	0.6440	0.6435	0.7130	0.7057	0.54134	0.53864	0.54134	0.53864
1500 m	0.6548	0.6539	0.7256	0.7235	0.55956	0.55705	0.55956	0.55705
1700 m	0.6742	0.6733	0.7341	0.7332	0.57654	0.57420	0.57654	0.57420
1900 m	0.6936	0.6846	0.7528	0.7442	0.59256	0.59033	0.59256	0.59033
2050 m	**0.7035**	**0.6940**	**0.7035**	**0.6940**	0.60400	0.60184	0.60400	0.60184
2500 m	0.7341	0.7332	0.7341	0.7332	0.63611	0.63417	0.63611	0.63417
3000 m	0.7639	0.7633	0.7639	0.7634	0.66866	0.66690	0.66866	0.66690
3500 m	0.7934	0.7928	0.7934	0.7934	0.69889	0.69718	0.69889	0.69718
4000 m	0.8238	0.8228	0.8238	0.8228	0.72712	0.72562	0.72712	0.72562
4500 m	0.8448	0.8441	0.8448	0.8448	0.75402	0.75254	0.75402	0.75254
5000 m	0.8730	0.8722	0.8730	0.8730	0.77959	0.77822	0.77959	0.77822

#### Case 6: single line-to-ground fault at t_f_ = 0.067 s

**Fig. 10:**
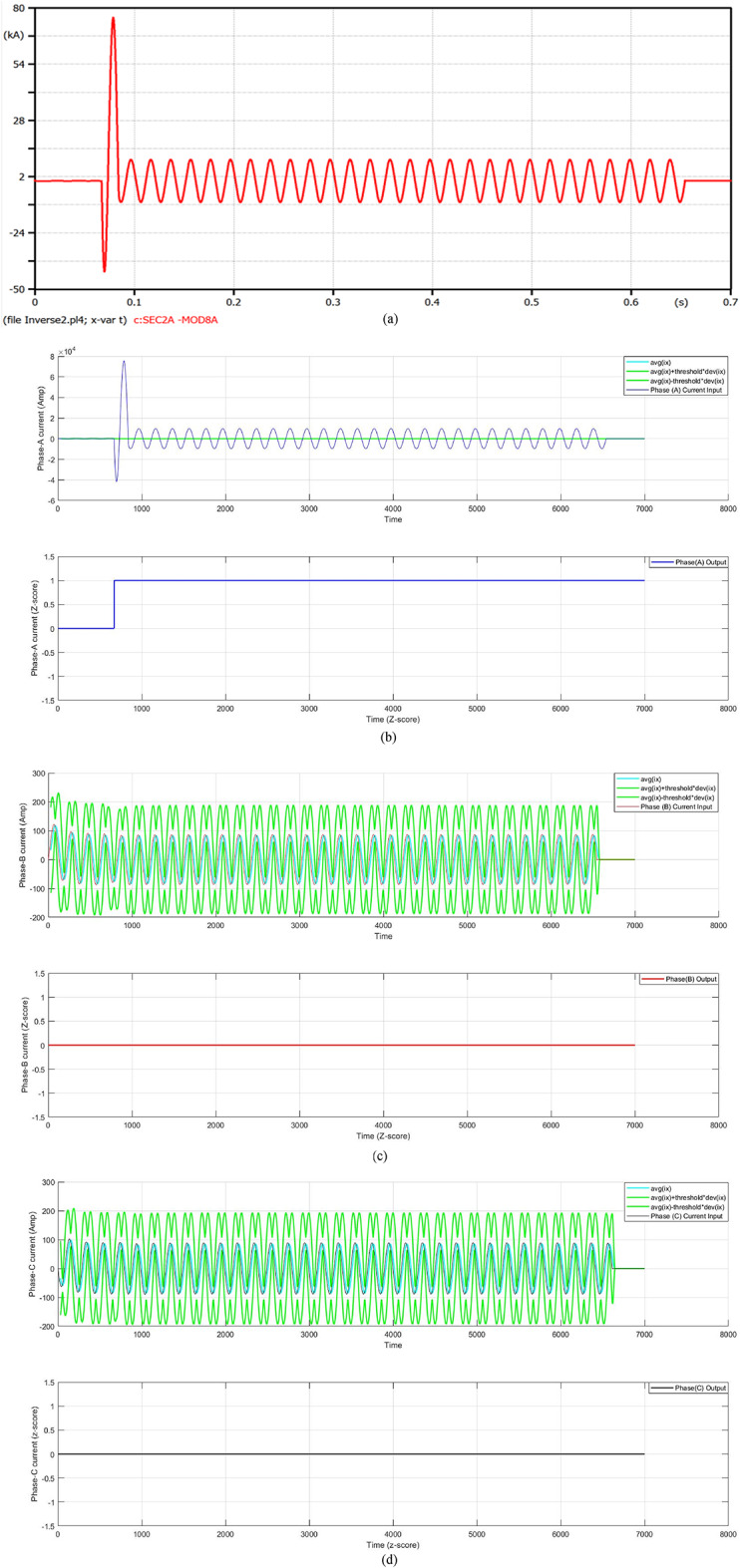
**a**–**d** Algorithm results in the case of a single line-to-ground fault at 0.067 s

In this case, a single line-to-ground fault happens on the distribution feeder at the transformer 2 at the time instant 0.067 s. Figure 10 presents a fault current with a fault distance equal to 100 m. The fault current increases to 75.43 kA after one cycle of the fault inception time, and the FCL suppresses the fault current. At that moment, the fault current is limited to 9.794 kA, but the COCR operating time (OPT) increases from 0.3670 s to 0.5939 s. In this instance, the proposed AOCR operating time is not affected by the FCL presence (i.e., it is fixed with or without the FCL), where the OPT is nearly 0.37646 s. Table 7 includes the values of the operating time for the conventional and adaptive OCRs for different locations (on the feeder cable) of single line-to-ground faults with *R*_*f*_ = 0.0 Ω at *t*_*f*_ = 0.067 s.

When using the conventional OCR at 100 m, the OPT is close to 0.3871 s in the case of without DG and without FCL, but it is 0.594 s in the case of without DG and with FCL. When using an adaptive OCR, the OPT value remains the same in both cases, where it equals about 0.39082 s. In addition, when using the conventional OCR at 100 m, the OPT is equal to 0.3670 s in the case of with DG and without FCL, but it is altered to 0.5939 s in the case of with DG and with FCL. When using an adaptive OCR, the OPT value remains the same in both cases, which is roughly 0.37646 s. For all scenarios of the SLGFs, the quantitative findings reveal that the AOCR is faster than the COCR, as shown in Table 7.

Table 7 includes the operating times (OPT) at disparate distances of the cable when the running load of the protected feeder is 100% of the base load (Z_L_ = j120.26 + j26.99 Ω) and a single line-to-ground fault (SLGF) condition with *R*_*f*_ = 0.0 Ω at *t*_*f*_ = 0.067 s. The operating times of the conventional and adaptive OCRs are quantified with/without FCLs and DGS.

**Table 7:** Operating time for single line-to-ground fault with *R*_*f*_ = 0.0 Ω at *t*_*f*_ = 0.067 s

Distance	Operating time (OPT) Single line-to-ground fault with Rf = 0.0 Ω at t_f_ = 0.067 s Z_L_ = j120.26 + j26.99 Ω							
	Conventional OCR				Adaptive OCR			
	Without FCL Without DGSM	Without FCL With DGSM	With FCL Without DGSM	With FCL With DGSM	Without FCL Without DGSM	Without FCL With DGSM	With FCL Without DGSM	With FCL With DGSM
100 m	0.3871	0.3670	0.5943	0.5939	**0.39082**	**0.37646**	**0.39082**	**0.37646**
300 m	0.4763	0.4752	0.6337	0.6334	0.44855	0.44026	0.44855	0.44026
500 m	0.5454	0.5445	0.6540	0.6443	0.49001	0.48377	0.49001	0.48377
700 m	0.5853	0.5850	0.6642	0.6633	0.52375	0.51858	0.52375	0.51858
900 m	0.5940	0.5922	0.6833	0.6828	0.55293	0.54839	0.55293	0.54839
1100 m	0.6246	0.6230	0.6949	0.6933	0.57898	0.57493	0.57898	0.57493
1300 m	0.6375	0.6362	0.7134	0.7130	0.60283	0.59910	0.60283	0.59910
1500 m	0.6638	0.6539	0.7242	0.7238	0.62492	0.62147	0.62492	0.62147
1700 m	0.6746	0.6742	0.7437	0.7346	0.64572	0.64246	0.64572	0.64246
1900 m	0.6940	0.6936	0.7533	0.7528	0.66539	0.66226	0.66539	0.66226
2050 m	**0.7039**	**0.7035**	**0.7039**	**0.7035**	0.67949	0.67655	0.67949	0.67655
2500 m	0.7341	0.7337	0.7341	0.7337	0.71938	0.71673	0.71938	0.71673
3000 m	0.7645	0.7634	0.7645	0.7634	0.76031	0.75778	0.76031	0.75778
3500 m	0.7949	0.7939	0.7949	0.7939	0.79842	0.79605	0.79842	0.79605
4000 m	0.8243	0.8233	0.8243	0.8233	0.83465	0.83240	0.83465	0.83240
4500 m	0.8532	0.8527	0.8532	0.8527	0.86919	0.86690	0.86919	0.86690
5000 m	0.8746	0.8741	0.8746	0.8741	0.90233	0.90010	0.90233	0.90010

### Different high impedances

#### Case 7: single line-to-ground fault with rf = 10 ohm

**Fig. 11:**
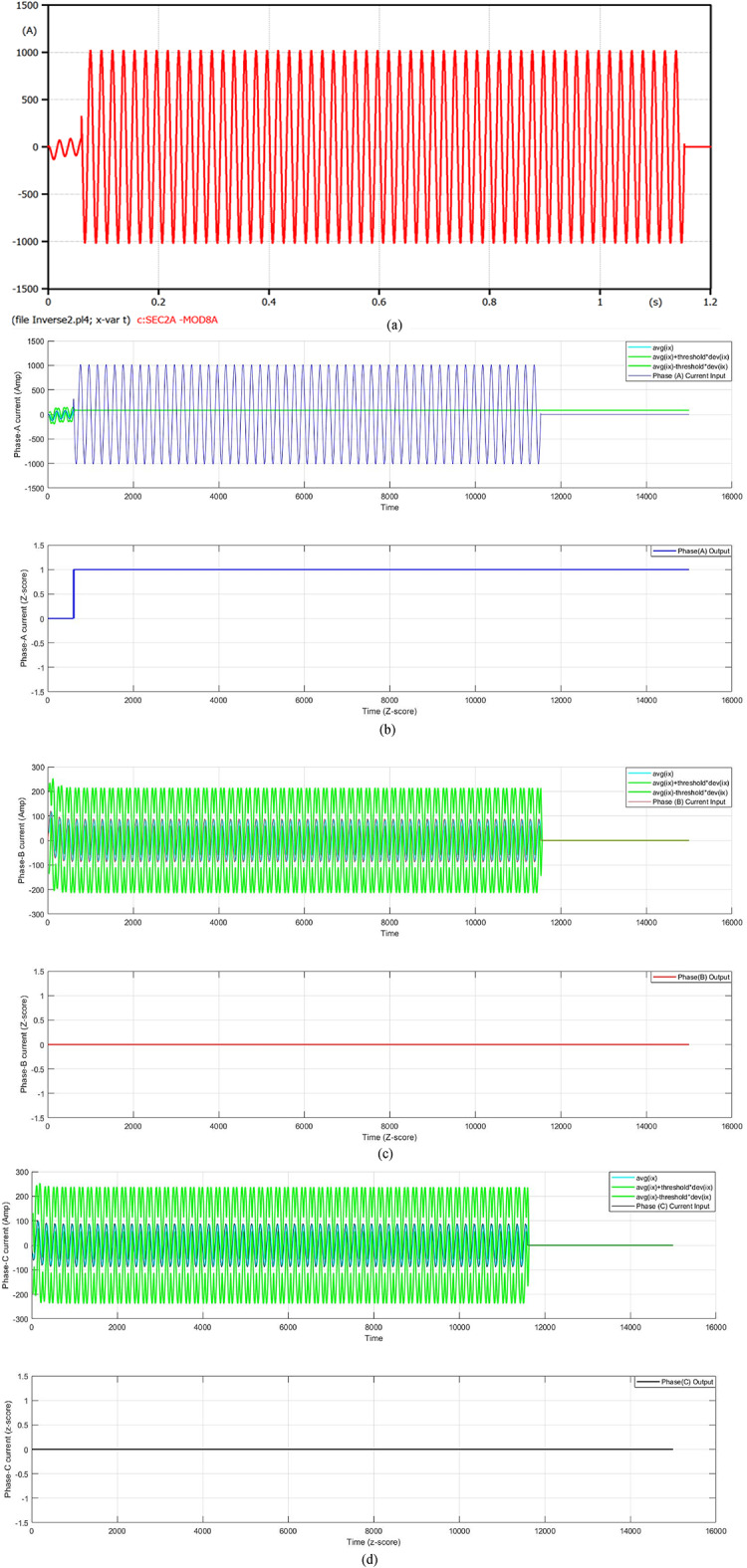
**a**–**d** Algorithm results in the case of a single line-to-ground fault through *R*_*f*_ = 10 Ω at 0.06 s

In this case, a single line-to-ground fault is located on the distribution feeder of the transformer 2 at the instant 0.06 s. A large fault resistance is inserted in the fault path, which is equal to 10.0 ohm. Figure 11 depicts the fault current in the case of the fault location equal to 1 km. The fault current evolves to 1.008 kA. The COCR operating time (OPT) is equal to 1.091 s. In this case study, the proposed AOCR operating time is not affected by the FCL entrance, where the OPT is equal to 0.98207 s with/without the FCL. Table 8 contains the values of the operating time for the conventional and adaptive OCRs for different locations (on the feeder cable) of single line-to-ground faults with *R*_*f*_ = 10.0 Ω at *t*_*f*_ = 0.060 s.

When using the conventional OCR at 1 km, the OPT is approximately 1.101 s in the case of without DG and without FCL, as well as in the case of without DG and with FCL. When using an adaptive OCR, the OPT value is the same in both cases, which equals 0.98280 s. Also, when using the conventional OCR at 100 m, the OPT is about 1.091 s in the case of with DG and without FCL, and in the case of with DG and with FCL. When using an adaptive OCR, the OPT value remains the same in both cases, which equals 0.98207 s. From the quantitative results in Table 8, it is obvious that the AOCR operating time is shorter than the COCR operating time for all instants of the SLGFs.

Table 8 presents the operating times (OPT) at diverse distances of the feeder cable when the running load of the protected feeder is 100% of the base load (Z_L_ = j120.26 + j26.99 Ω) and a single line-to-ground fault (SLGF) condition with *R*_*f*_ = 10 Ω at *t*_*f*_ = 0.06 s. The operating times of the conventional and adaptive OCRs are calculated with/without FCLs and DGS.

**Table 8:** Operating time for single line-to-ground fault with *R*_*f*_ = 10 Ω at *t*_*f*_ = 0.06 s

Distance	Operating time (OPT) Single line-to-ground fault with Rf = 10 Ω at t_f_ = 0.06 s Z_L_ = j120.26 + j26.99 Ω							
	Conventional OCR				Adaptive OCR			
	Without FCL Without DGSM	Without FCL With DGSM	With FCL Without DGSM	With FCL With DGSM	Without FCL Without DGSM	Without FCL With DGSM	With FCL Without DGSM	With FCL With DGSM
1000 m	1.101	1.091	1.101	1.091	0.98280	0.98207	0.98280	0.98207
2000 m	1.132	1.130	1.132	1.130	1.02060	1.01980	1.02060	1.01980
3000 m	1.172	1.170	1.172	1.170	1.05790	1.05740	1.05790	1.05740
4000 m	1.211	1.210	1.211	1.210	1.09530	1.09410	1.09530	1.09410
5000 m	1.251	1.250	1.251	1.250	1.1314	1.13008	1.1314	1.13008

#### Case 8: single line-to-ground fault with Rf = 50 Ω

Table 9 contains the operating times (OPT) at disparate distances of the feeder cable when the running load of the protected feeder is 100% of the base load (Z_L_ = j120.26 + j26.99 Ω) and a single line-to-ground fault (SLGF) condition with *R*_*f*_ = 50 Ω at *t*_*f*_ = 0.06 s. The operating times of the conventional and adaptive OCRs are computed with/without FCLs and DGS.

**Table 9:** Operating time for single line-to-ground fault with *R*_*f*_ = 50 Ω at *t*_*f*_ = 0.06 s

Distance	Operating time (OPT) Single line-to-ground fault with *R*_f_ = 50 Ω at t_f_ = 0.06 s Z_L_ = j120.26 + j26.99 Ω							
	Conventional OCR				Adaptive OCR			
	Without FCL Without DGSM	Without FCL With DGSM	With FCL Without DGSM	With FCL With DGSM	Without FCL Without DGSM	Without FCL With DGSM	With FCL Without DGSM	With FCL With DGSM
1000 m	2.528	2.516	2.528	2.516	2.3551	2.3551	2.3551	2.3551
2000 m	2.578	2.576	2.578	2.576	2.4062	2.4062	2.4062	2.4062
3000 m	2.648	2.638	2.648	2.638	2.4515	2.4515	2.4515	2.4515
4000 m	2.710	2.692	2.710	2.692	*2.4993*	*2.4895*	*2.4993*	*2.4895*
5000 m	2.767	2.751	2.767	2.751	2.5396	2.5396	2.5396	2.5396

#### Case 9: single line-to-ground fault with rf = 100 Ω

Table 10 includes the operating times (OPT) at various distances of the feeder cable when the running load of the protected feeder is 100% of the base load (Z_L_ = j120.26 + j26.99 Ω) and a single line-to-ground fault (SLGF) condition with *R*_*f*_ = 100 Ω at *t*_*f*_ = 0.06 s. The operating times of the conventional and adaptive OCRs are estimated with/without FCLs and DGS.

The algorithm is validated for SLGFs with *R*_*f*_ = 10 Ω, *R*_*f*_ = 50 Ω, and *R*_*f*_ = 100 Ω, as demonstrated in Tables 8 and 9, and 10, respectively. These changes do not affect the technique’s effectiveness, except that its operating time values become larger as the *R*_*f*_ values increase. Therefore, the developed technique is effective and efficient for earth faults that typically involve high fault resistances, where the data window size is selected one cycle of the fundamental frequency.

**Table 10:** Operating time for single line-to-ground fault with *R*_*f*_ = 100 Ω at *t*_*f*_ = 0.06 s

Distance	Operating time (OPT) Single line-to-ground fault with *R*_f_ = 100 Ω at t_f_ = 0.06 s Z_L_ = j120.26 + j26.99 Ω							
	Conventional OCR				Adaptive OCR			
	Without FCL Without DGSM	Without FCL With DGSM	With FCL Without DGSM	With FCL With DGSM	Without FCL Without DGSM	Without FCL With DGSM	With FCL Without DGSM	With FCL With DGSM
1000 m	4.778	4.745	4.778	4.745	4.4454	4.4454	4.4454	4.4454
2000 m	4.918	4.884	4.918	4.884	4.4931	4.4931	4.4931	4.4931
3000 m	5.072	5.025	5.072	5.025	4.5927	4.5927	4.5927	4.5927
4000 m	5.219	5.176	5.219	5.176	4.6980	4.6980	4.6980	4.6980
5000 m	5.380	5.327	5.380	5.327	4.7530	4.7530	4.7530	4.7530

### Operating load changes with/without faults

#### Operating load change with fault condition (SLGF)

Table 11 shows the operating times (OPT) at various distances of the feeder cable when the running load of the protected feeder changes with 80% of the base load (Z_L_ = j96.205 + j21.59 Ω) and a single line-to-ground fault (SLGF) condition with *R*_*f*_ = 0.0 Ω at *t*_*f*_ = 0.06 s. The operating times of the conventional and adaptive OCRs are estimated with/without FCLs and DGS.

**Table 11:** Operating time for 80% load change and single line-to-ground fault with *R*_*f*_ = 0.0 Ω at *t*_*f*_ = 0.06 s

Distance	Operating time (OPT) 80% Load change with single line-to-ground fault with *R*_f_ = 0.0 Ω at t_f_ = 0.06 s Z_L_ = j96.205 + j21.59 Ω							
	Conventional OCR				Adaptive OCR			
	Without FCL Without DGSM	Without FCL With DGSM	With FCL Without DGSM	With FCL With DGSM	Without FCL Without DGSM	Without FCL With DGSM	With FCL Without DGSM	With FCL With DGSM
100 m	0.3469	0.3265	0.5636	0.5534	**0.36905**	**0.35699**	**0.36905**	**0.35699**
1100 m	0.5741	0.5739	0.6530	0.6525	0.54282	0.53961	0.54282	0.53961
2100 m	**0.6634**	**0.6629**	**0.6634**	**0.6629**	0.63624	0.63391	0.63624	0.63391
3500 m	0.7533	0.7528	0.7533	0.7528	0.73614	0.73437	0.73614	0.73437
5000 m	0.8329	0.8324	0.8329	0.8324	0.82567	0.82419	0.82567	0.82419

Table 12 illustrates the operating times (OPT) at different distances of the feeder cable when the running load of the protected feeder changes with 120% of the base load (Z_L_ = j144.31 + j32.38 Ω) and a single line-to-ground fault (SLGF) condition with *R*_*f*_ = 0.0 Ω at *t*_*f*_ = 0.06 s. The tripping times of the traditional and adaptive OCRs are quantified with/without FCLs and DGS. The performance of the proposed algorithm has been examined under both large load changes (within ± 20%) with single line-to-ground faults (SLGFs), as shown in Tables 11 and 12, respectively. These changes do not affect the technique’s effectiveness and efficiency.

**Table 12:** Operating time for 120% load change and single line-to-ground fault with *R*_*f*_ = 0.0 Ω at *t*_*f*_ = 0.06 s

Distance	Operating time (OPT) 120% Load change with single line-to-ground fault with *R*_f_ = 0.0 Ω at t_f_ = 0.06 s Z_L_ = j144.31 + j32.38 Ω							
	Conventional OCR				Adaptive OCR			
	Without FCL Without DGSM	Without FCL With DGSM	With FCL Without DGSM	With FCL With DGSM	Without FCL Without DGSM	Without FCL With DGSM	With FCL Without DGSM	With FCL With DGSM
100 m	0.3876	0.3469	0.5738	*0.5937*	**0.34510**	**0.33444**	**0.34510**	**0.33444**
1100 m	0.6143	0.6040	0.6931	0.6929	0.49454	0.49185	0.49454	0.49185
2100 m	**0.7044**	**0.7035**	**0.7044**	**0.7035**	0.57201	0.57012	0.57201	0.57012
3500 m	0.7939	0.7918	0.7939	0.7918	0.65293	0.65148	0.65293	0.65148
5000 m	0.8735	0.8718	0.8735	0.8718	0.72389	0.72268	0.72389	0.72268

#### **Operating load change without any fault condition**

Table 13 presents the operating times (OPT) at different distances of the cable when the running load of the protected feeder changes with 120% of the base load (Z_L_ = j144.31 + j32.38 Ω) without any fault condition. The operating times of the conventional and adaptive OCRs are recorded with/without FCLs and DGs. The performance of the proposed algorithm has been examined under load changes within + 20% of the base load. These changes do not affect the technique’s performance. Thus, the developed technique is robust against the load changes, as illustrated in Table 13. It is observed that the operating time (OPT) is infinite for both COCR and AOCR with/without FCLs and DGs.

**Table 13:** Operating time for 120% load change without any fault condition

Distance	Operating time (OPT) 120% Load change without any fault condition Z_L_ = j144.31 + j32.38 Ω							
	Conventional OCR				Adaptive OCR			
	Without FCL Without DGSM	Without FCL With DGSM	With FCL Without DGSM	With FCL With DGSM	Without FCL Without DGSM	Without FCL With DGSM	With FCL Without DGSM	With FCL With DGSM
100 m	∞	∞	∞	∞	∞	∞	∞	∞
1100 m	∞	∞	∞	∞	∞	∞	∞	∞
2100 m	∞	∞	∞	∞	∞	∞	∞	∞
3500 m	∞	∞	∞	∞	∞	∞	∞	∞
5000 m	∞	∞	∞	∞	∞	∞	∞	∞

In this study, the quantitative findings reveal the following observations:

1. The proposed scheme is effective for single-phase faults (SLGF) and multi-phase faults (DLF, DLGF, and 3LF), as illustrated in Tables 2, 3 and 4, and 5, respectively.
2. It is also effective for single line-to-ground faults (SLGFs) at different fault inception times, such as *t*_*f*_ = 0.060 s, *t*_*f*_ = 0.063 s, and *t*_*f*_ = 0.067 s, as shown in Tables 2 and 6, and 7, respectively.
3. It is also effective for earth faults that involve high fault resistances, such as *R*_*f*_ = 10.0 Ω, *R*_*f*_ = 50.0 Ω, and *R*_*f*_ = 100.0 Ω, as demonstrated in Tables 8 and 9, and 10, respectively.
4. The performance of the proposed algorithm has been examined under both large load changes (within ± 20%) and single line-to-ground faults (SLGFs), as shown in Tables 11 and 12, respectively. These changes do not affect the technique’s effectiveness and efficiency.
5. The developed technique is robust against the load changes (within ± 20%), as given in Table 13.
6. The proposal is superior to the conventional OCRs for many protection characteristics, such as operating speed, sensitivity, reliability, and adaptive characteristic curve.
7. The advanced algorithm can function online and adapt its settings automatically according to the level of the fault current and operating load.
8. The Z-score for each phase current signal can be used to select the faulty phase, and define a fault type using the three Z-scores of the three-phase current signals measured using the proposed AOCR.
9. The proposed scheme is applicable to various distribution systems with/without FCLs and DGs.

##  Critical comparison

As illustrated in Table 14, a comparison is held between the proposed adaptive OCR based on the Z-score and the Mean Ratio (MR) of the phase current and other conventional protective relays. This work is advantageous, as it is similar to the method presented in^39^, but the present method is the fastest and the simplest.

**Table 14:** Comparison between the proposed adaptive OCR and conventional protective relays

Item	Proposed adaptive OCR	Conventional protective relays
1. Input analog signals	It uses only the three-phase currents of the power system component, and it does not require more inputs like voltage, FCL impedance, or other factors.	Some conventional OCRs need other analog inputs, like voltage^11,24,34,35,40,43–47^ or FCL impedance^26,27,29–31,33,34,36^
2. Basic criteria	It relies on the current Mean Ratio (MR), which is calculated per each data window selected in between one cycle and a sub-cycle. Therefore, the relay sensitivity is controllable using the selected data set, and the relay has the ability to operate at different sizes of data window.	They rely on the RMS value estimated for each cycle^11,14,16,24^
3. Mathematical formulas	It uses the same mathematical formula (for both earth fault and phase overcurrent protections) to calculate the operating time for both earth fault and phase overcurrent relays.	They apply different mathematical formulas for calculating the operating time for both earth fault and phase overcurrent relays^43,45,47^
4. Relay operation	It operates automatically and online in various power systems with different ratings. As a result of being non-selected, the algorithm can be named an automated protection system, which does not require any change of setting group in the protection relay.	It does not represent an automated protection system, and it requires some changes in the relay settings^11,15,19,24,42–45,47^
5. Relay setting	It does not require entering pickup current that can be modified with different operating conditions, such as light/full loading, and high impedance.	It needs to select manually proper settings of the pickup current/voltage^40–47^. The relay settings are not adaptive^40–47^
6. Adaptation	It automatically adjusts the relay protection settings in response to the changes in the power network status.	Both relays (with fixed protection settings) are not suitable for changes in the power network status, especially after the widespread and increasing penetration of the DGs in the networks^1,3,4,9,11,13,16,19,20,29^
7. Relay attributes	It quantifies the improvements in terms of dependability, security, sensitivity, and operating time, presented by the adaptive overcurrent system over traditional systems.	The fixed settings affect the protection properties, such as dependability, security, sensitivity, and operating time^1,3,4,9,11,13,16,18,20,29,30,36^
8. Relay sensitivity	The Mean Ratio (MR), which is based on the level of loading and short-circuit currents, respectively, is adaptive. As a result, the MR controls the relay sensitivity.	The pickup current of the phase overcurrent/ground fault current relay is a constant value^14,15,28,29,40^
9. Relay speed	The developed approach is simple and fast.	Some existing approaches are more complicated^4,7,13,18,43,47^ and slower^11,13,15,25,34,39,43–45,47^
10. Relay stability	It is stable under various non-ideal operating conditions, such as different load changes, descent ripples, DC components, and measurement errors of current transformers. Noise immunity and current filtering can be accomplished in this study due to applying the data window concept. It operates for different fault types, such as ground or phase faults, with/without or and with/without DGs.	Some protection methods lose their stability under different non-ideal operating conditions (with/without FCLs, or with/without DGs)^4,11,14,37^
11. Faulty phase selection	In this technique, the Z-score of the phase current signal is able to specify the faulty phase and categorize the fault type.	Several existing approaches are unable to define the faulty phase or the fault type^42–44^

##  Conclusions

This article has presented an adaptive OCR based on the Z-score and the Mean Ratio (MR) for each phase current signal, which is considered a proper solution against comprehensive fault scenarios. It has been investigated under different fault types, fault inception angles, and high fault resistances with/without FCLs and DGs. It can be characterized by high reliability in all fault scenarios for DGs, where it can avert the problem of the time delay that happens with the conventional OCR during the FCL entrance to the distribution network. The present algorithm does not require changing the protection relay settings for any possible fault scenario; it is independent on the impedance value of the FCL, and it does not affect the relay operating time. Besides, it has continuous stability against all fault types, accompanied by the FCLs that limit the short-circuit currents. Moreover, the algorithm only needs the three-phase currents to compute their Z-score that has been used to define any possible fault, and they have been used to quantify their Mean Ratios (MRs) that have been applied to estimate the suitable tripping time of the proposed OCR. Comprehensive examinations have revealed that the proposal is verified under the influence of load changes and different types of shunt faults, fault inception times, and fault resistances with/without FCLs and with/without DGs. The simulation results have demonstrated that the proposed solution is superior to the traditional OCRs for multiple protection characteristics, such as operation speed, adaptation stability, and sensitivity. The quantitative findings confirm that the protection algorithm can run immediately and modify its settings automatically in accordance with the fault and operating situations. As a result, it has the ability to find fault instances upon which the protection issues a tripping signal to the circuit breakers of the intended protection zone, but it stays silent under no-fault conditions. The algorithm speed and sensitivity can be altered using the size of the data set. Moreover, it is described as being easy to employ, precise, and reliable. Thus, the approach can be amalgamated with other digital protection systems to protect traditional and smart grids, as well as digital substations.

## Electronic supplementary material

Below is the link to the electronic supplementary material.

Supplementary Material 1

## Data Availability

All data generated or analysed during this study are included in this published article [and its supplementary information files].
