# Supplementary material for: Adaptive overcurrent protection considering fault current limiters effect
Source: Sci Rep. 2025 Jun 20;15:20146. doi: 10.1038/s41598-025-05135-5 (PMC12181245; doi:10.1038/s41598-025-05135-5)
Supplement: Supplementary file 1 — Supplementary Material 1 [file 41598_2025_5135_MOESM1_ESM.pdf]

Nomenclatures:

Symbols	Abbreviations
DGs	Distributed Generators
FCLs	Fault Current Limiters
OCRs	Overcurrent Relays
MR	Mean Ratio
ATP	Alternative Transient Program
Isc	Short-circuit Current
Ip	Pick-up Current
CLR	Current Limiting Resistor/Reactor
DERs	Distributed Energy Resources
TD	Time Dial
OPT	the Relay Operating Time
SFCLs	Superconducting Fault Current Limiter
CTI	Coordination Time Interval
CB	Circuit Breaker
**COCRs**	Conventional Overcurrent Relays
**AOCRs**	Adaptive Overcurrent Relays
**Istd**	Standard Deviation of Current Signal
**SLGF**	Single Line-to-Ground Fault
**DLGF**	Double Line-to-Ground Fault
DLF	Double Line Fault
3LGF	Three Line-to-Ground Fault
3LF	Three Line Fault
R_f	Fault Resistance (in Ω)
t_f	Fault Inception Time (in Sec)

Appendix 1: The parameters' data of the simulated power system components

**Parameters of power system components**	**Data**
**Synchronous generator (Connected to feed 220 kV network):**	
Rated Volt-ampere	*192 MVA*
Rated line voltage	*18 kV*
Voltage phasor angle	*9.3^0*
Rated frequency	*50 Hz*
Number of poles	*2*
**Main Transformer: (18 kV/220 kV)**	
Rated Volt-ampere	*100 MVA*
Transformation voltage ratio	*18 kV /220 kV*
Connection primary/secondary	*Delta/Star earthed neutral*
Vector group	*D-YN11*
Z%	*6.25 %*
**Main Transformer: (220 kV/66 kV)**	
Rated Volt-ampere	*40 MVA*
Transformation voltage ratio	*220 kV /66 kV*
Connection primary/secondary	*Star/Star earthed neutral*
Vector group	*YN-YN0*
Z%	*8 %*
**Synchronous generator (Connected to feed 66 kV network):**	
Rated Volt-ampere	*192 MVA*
Rated line voltage	*18 kV*
Voltage phasor angle	*9.3^0*
Rated frequency	*50 Hz*
Number of poles	*2*
**Main Transformer: (18 kV/66 kV)**	
Rated Volt-ampere	*100 MVA*
Transformation voltage ratio	*18 kV /66 kV*
Connection primary/secondary	*Delta/Star earthed neutral*

Vector group<br><br>Z%	*D-YN11*<br><br>*7 %*
**Main Transformer: (66 kV/11 kV)**<br><br>Rated Volt-ampere<br><br>Transformation voltage ratio<br><br>Connection primary/secondary<br><br>Vector group<br><br>Z%	 <br>*25 MVA*<br><br>*66 kV /11 kV*<br><br>*Delta/Star earthed neutral*<br><br>*D-YN11*<br><br>*11 %*
**Electrical Load: (mod8)**<br><br>Active power<br><br>Reactive power<br><br>Power factor	 <br>10 MW<br><br>7 MVAR<br><br>0.82
**Medium Voltage (MV) feeder cable:**<br><br>Resistance<br><br>Inductance<br><br>Capacitance	 <br>0.0009271 Ohm/m<br><br>0.0005289 Ohm/m<br><br>0.0002094 µf/m
**Current Transformers: (mod8)**<br><br>CTR<br><br>Rated burden<br><br>Class	 <br>*600/5 A*<br><br>*30 VA*<br><br>*5p20*

Appendix 2: Input quantities for the protection algorithm

Quantity designation	Quantity description	Numerical value
ΔT_s	Sampling time interval of the digital system	0.0001 Sec
F_n	Fundamental frequency of the power system	50 Hz
F_s	Sampling frequency of the digital system	10 kHz
l	Lag (the algorithm will react to the pattern once every n-samples)	30 samples
I_n	Influence (If (0) denotes that the data has no impact on the threshold (that assumes a stable process))	0 %
th	Threshold (The algorithm detects the points which are outside this threshold as peaks)	7 Istd
i^{smooth}	Smoothed Input Signal	y = $[y^1, y^2, \ldots, y^N]$,
μ	Moving Mean from a smoothed signal(i^{smooth}).	$\bar{\mu}_i = \frac{1}{l}\sum_{i}^{i+l} \mu_i$
σ	Moving Standard Deviation from a smoothed signal(i^{smooth}).	$\sigma_{\mu_i} = \sqrt{\frac{\sum_i^{i+l}(\mu_i - \bar{\mu}_i)^2}{l-1}}$
Z-score peak	Detection method analyzes the signal's evolution using a changing mean and calculates standard deviations to establish a threshold around the signal	$z_i = \frac{x_i - \bar{\mu}_{i-1}}{\sigma_{\mu_{i-1}}}$
MR_m	The measured value is the current mean ratio at the present cycle	$MR = \frac{I_{avg})_n}{I_{avg})_{n-Nc}}$
MR_s	The setting value of the current mean ratio is adapted by calculating the mean ratio at the previous cycle	$MR = \frac{I_{avg})_n}{I_{avg})_{n-Nc}}$
$I_{avg})_n$	The average current for a present cycle	Calculated by algorithm
$I_{avg})_{n-Nc}$	The average current for the previous cycle	Calculated by algorithm
OPT	Relay operating time	$t = TMS \frac{A}{\left(\frac{I_{avg})_n}{I_{avg})_{n-Nc}}\right)^B - 1}$
A	A is from 0 to 2 (with step 0.01)	A = 0.02
B	B is from 0 to 15 (with step 0.01)	B = 0.14
TMS	TMS = Time Multiplier Setting is from 0.05 to 1 (with step 0.01)	TMS = 0.35

Z_L	The load impedance of the feeder required to be protected	Z_L = j120.26 + j26.99
